# The interplay of polarizable water and protein in the activation of the M2 channel

**DOI:** 10.3389/fphar.2025.1532697

**Published:** 2025-09-02

**Authors:** Márta Gődény, Nóra Kovács, Aamir Aman, Thanyada Rungrotmongkol, Christian Schröder

**Affiliations:** ^1^ Department of Computational Biological Chemistry, University of Vienna, Vienna, Austria; ^2^ Vienna Doctoral School in Chemistry (DoSChem), University of Vienna, Vienna, Austria; ^3^ Institut für Chemie, Theoretische Chemie, Technische Universität, Berlin, Germany; ^4^ Center of Excellence in Structural and Computational Biology, Department of Biochemistry, Faculty of Science, Chulalongkorn University, Bangkok, Thailand; ^5^ Program in Bioinformatics and Computational Biology, Graduate School, Chulalongkorn University, Bangkok, Thailand

**Keywords:** influenza a virus, M2 proton channel, polarizability, channel gating dynamics, molecular dynamics simulations

## Abstract

The M2 proton channel within the Influenza A virus constitutes an essential element for viral replication, with its functionality depending on the protonation states of four histidine residues located within the channel. This study meticulously investigates the impact of polarizability on the channel’s gating dynamics across various protonation states, employing both polarizable and non-polarizable models for water, protein, and membrane. Through a comprehensive analysis, we elucidate the nuanced role of polarizability in the channel’s operational mechanisms, differentiating between the solvent and protein polarizable effects. This investigation not only enriches our understanding of the M2 channel’s biophysical behavior but also highlights the significance of polarizability.

## 1 Introduction

The Influenza A virus is a major pathogen responsible for seasonal influenza epidemics. It critically depends on the M2 proton channel for replication. Thus, the channel’s transmembrane segment is a pivotal target for developing antiviral drugs, spurring extensive investigative efforts to decode its proton transfer mechanisms. A seminal study by Hu et al. (2010) employed solid-state NMR techniques to delineate a proton conduction mechanism, unveiling the structural and functional impacts of pH variations on the channel. At elevated pH levels, the formation of CH- π stacks by neutral histidine residues obstructs establishing an H-bonded aqueous chain. Conversely, lower pH conditions induce protonation, leading to imidazolium formation, pore expansion, and enhanced water penetration and proton transfer, facilitated by dynamic microsecond rotations of imidazolium rings. Subsequent work by Hu et al. (2011) utilized magic-angle-spinning solid-state NMR spectroscopy to illuminate His37-water interactions, highlighting the moderating influence of Trp41 on proton exchange rates and challenging prevailing models of proton conduction within the M2 channel. Hong et al. (2012) furthered the discourse on His37 interaction mechanisms through solid-state NMR, evidencing His37’s conventional hydrogen bonding with water, thereby supporting the His-water proton exchange model. Dyer’s innovative approach (Jeong and Dyer, 2017), combining laser-induced pH jumps with time-resolved fluorescence spectroscopy, observed the rapid protonation of His37 and ensuing structural alterations, hinting at Asp24 residues’ role in proton gathering and questioning the M2 channel’s transporter-like mechanism. More recently, Fu et al. (2020) applied two-dimensional J-resolved NMR spectrum analysis to study the His37 tetrad within the M2 protein, confirming the existence of diverse imidazole-imidazolium hydrogen bonds and endorsing the low-barrier hydrogen bond mechanism for proton conductance, while suggesting improvements for local structural models across various contexts.

A comprehensive series of molecular dynamics (MD) and quantum mechanics/molecular mechanics (QM/MM) simulations conducted by Jorgensen et al. (1983), Zhong et al. (1998), Khurana et al. (2009), and Carnevale et al. (2010) have shed light on the intricate dynamics of proton transport and the structural integrity of the M2 proton channel across a spectrum of pH levels. In particular, Carnevale et al. (2010) used classical and hybrid QM/MM simulations to study different pH conditions, focusing on the role of water molecules and the four His37 residues in the proton transport mechanism. These simulations revealed distinctive configurations of water molecules within the channel, forming layered structures through strong hydrogen bonding, serving as temporary proton storage sites. Such configurations suggest a conducive free-energy landscape for translocating a classical hydronium-like entity, pinpointing specific regions within the channel that favor cation diffusion. Further investigations have examined the behavior of the channel under various protonation states, with a particular focus on the interactions between water molecules and histidine residues. These interactions profoundly impact the channel’s conformation and proton conduction capabilities. For instance, it was demonstrated (Wei and Pohorille, 2013) that the channel remains inert in the 0, +1, and +2 protonation states of the His37 residues and becomes active only upon further protonation, leading to the +3 and +4 states, attributable to electrostatic repulsion among the protonated histidines which modulates the proximity of adjacent protein α -helix structures. In subsequent years, Klein and co-workers (Dong et al., 2013; 2014) expanded their exploration of the M2 channel through QM/MM simulations, validating the stability of diverse His37 tetrad models at neutral pH. These models advocate for a multifaceted proton conduction paradigm, allowing the channel to stabilize two protons in various configurations. Biased simulations, such as multiscale reactive MD, have successfully provided free energy profiles that elucidate proton transport pathways (Liang et al., 2014; 2016; Watkins et al., 2019), demonstrating their strength in capturing thermodynamics. Recently, Voth and co-workers (Kaiser et al., 2024) reported on GPU acceleration of multiscale simulations using the RAPTOR package in LAMMPS, enabling the generation of significantly longer trajectories than those typically feasible with QM/MM. These free energy simulations and unbiased simulations are designed for different objectives: while biased methods are specifically designed to determine free energy barriers by carefully chosen collective variables, unbiased simulations target straightforward dynamical properties like diffusivity, e.g., proton diffusion coefficients, but may miss important rare events.

Conventional MD simulations may cover timescales for dynamical processes, however, at the cost of disregarding transient protonation of the histidine residues and the proton hopping in the channel water molecules. Constant pH MD simulations (Chen et al., 2016) have provided critical insights into the $pK_{a}$ values of the His37 tetrad and the thermodynamics of its protonation, thereby clarifying the proton conduction at the tetrad across different protonation states. They have also been employed to investigate other proton-gated ion channels (Jansen et al., 2024). Even some water molecules can be made titrable in constant pH simulations (Chen et al., 2013). Fully simulating proton conduction in the M2 channel of influenza A demands accounting for multiple protonation events in both the histidine tetrad and the network of water molecules lining the pore. Such proton hopping among water molecules remains beyond the scope of conventional constant-pH frameworks, which would require vastly increasing the number of protonable sites (potentially hundreds).

We have developed a Python-based program, Protex (Joerg et al., 2023; Gődény et al., 2024), which efficiently handles hundreds of simultaneous proton transfers between water molecules and between water and histidine residues during polarizable MD simulations without incurring significant computational overhead (Joerg et al., 2023). This capability enables detailed investigation of the Grotthuss mechanism in the channel, as consecutive proton-hopping events can be monitored over trajectories spanning several hundreds of nanoseconds. Currently, Protex can be applied in combination with OpenMM (Eastman et al., 2017) using CHARMM (Brooks et al., 2009) force field files. Polarizable forces are a prerequisite for our pseudo-reactive simulations, as they smooth the transient Coulomb energy by reacting to changes in the local electric field when molecules get protonated or deprotonated. Water molecules approaching a histidine residue affect the electronic distribution of the amino acid, which may stabilize its solvation. Also, the molecular water dipole increases in this environment (Dang, 1998; Devereux and Popelier, 2007). Consequently, we expect stronger hydrogen bonds using polarizable force fields. However, this does not only apply to hydrogen bonds between histidine and water but also between two histidines. The current study analyzes the effect of polarizability on the stability of the M2 ion channel at various protonation states. We also dissect the effect of the polarizable solvent water and the polarizable protein. This knowledge is fundamental for a subsequent study applying Protex (Joerg et al., 2023; Gődény et al., 2024) since we can then discuss the effect of proton hopping in addition to the effects of polarizability. The free energy profiles obtained from multiscale simulations (Liang et al., 2014; 2016; Watkins et al., 2019) serve as a valuable tool for determining reaction probabilities, which are essential for parameterizing the reaction kinetics in Protex.

One of the commercial drugs used to treat influenza that functions by blocking the M2 channel is amantadine (Oxford and Galbraith, 1980). Our previous MD simulations (Intharathep et al., 2008) indicated two preferred binding positions of amantadine. The first one was deep into the channel, close to the His37 tetrad. This position can lead to a more effective inhibition, as it can directly hinder the protonation of histidines, thus keeping the channel closed. The other binding site was closer to the opening of the channel. In this case, amantadine can block the transport through the channel by physically not letting water molecules pass.

## 2 Methods

### 2.1 Setup for the M2 channel

We investigated the M2 channel embedded in a 1-palmitoyl-2-oleoylphosphatidylcholin (POPC) membrane using different protonation states of the His37 tetrad, while also varying which parts of the system are described with a polarizable or non-polarizable force field. In addition to an “empty” channel (i.e., a channel filled only with water), we also investigated the effect of an inhibitor (amantadine). The resulting ion channel systems are summarized in Table 1. In the case of the protonation state +2, two independent simulations were performed: one with adjacent protonated histidines (HSP) (2Ha) and one simulation where the two HSPs are opposite of each other (diagonal, 2Hd). Furthermore, three independent replicas for each combination of membrane/protein, water, and protonation state were simulated, and their results were averaged to increase the statistics. This resulted in 72 independent simulations each for the systems with and without the inhibitor as summarized in Table 1.

**TABLE 1:** Overview of the simulated systems.

System			Protonation states
Membrane	Protein	Water	
non-pol	non-pol	non-pol	0, +1, +2a, +2d, +3, +4
non-pol	non-pol	pol	0, +1, +2a, +2d, +3, +4
pol	pol	non-pol	0, +1, +2a, +2d, +3, +4
pol	pol	pol	0, +1, +2a, +2d, +3, +4

The different protonation states were created by changing the residue type of the corresponding number of His37 from HSD (neutral) to HSP (positively charged). a and d describe the adjacent or diagonal protonation of the histidine residues, respectively. We performed three independent simulations for each membrane/protein, water, and protonation state combination.

We set up these systems using the membrane builder function (Jo et al., 2007; Wu et al., 2014) of CHARMM-GUI (Jo et al., 2008; Lee et al., 2016), starting directly from the PDBs described below. Only the four transmembrane chains and, if applicable, amantadine were selected from the PDB to build the system. Other molecules (e.g., water, ions, additional copies of the helices) were disregarded. Hydrogen coordinates and patching of the termini (NTER: positively charged N terminus and CTER: negatively charged C terminus) were added by CHARMM-GUI. No external preprocessing was conducted on the PDB structures.

**FIGURE 1:**
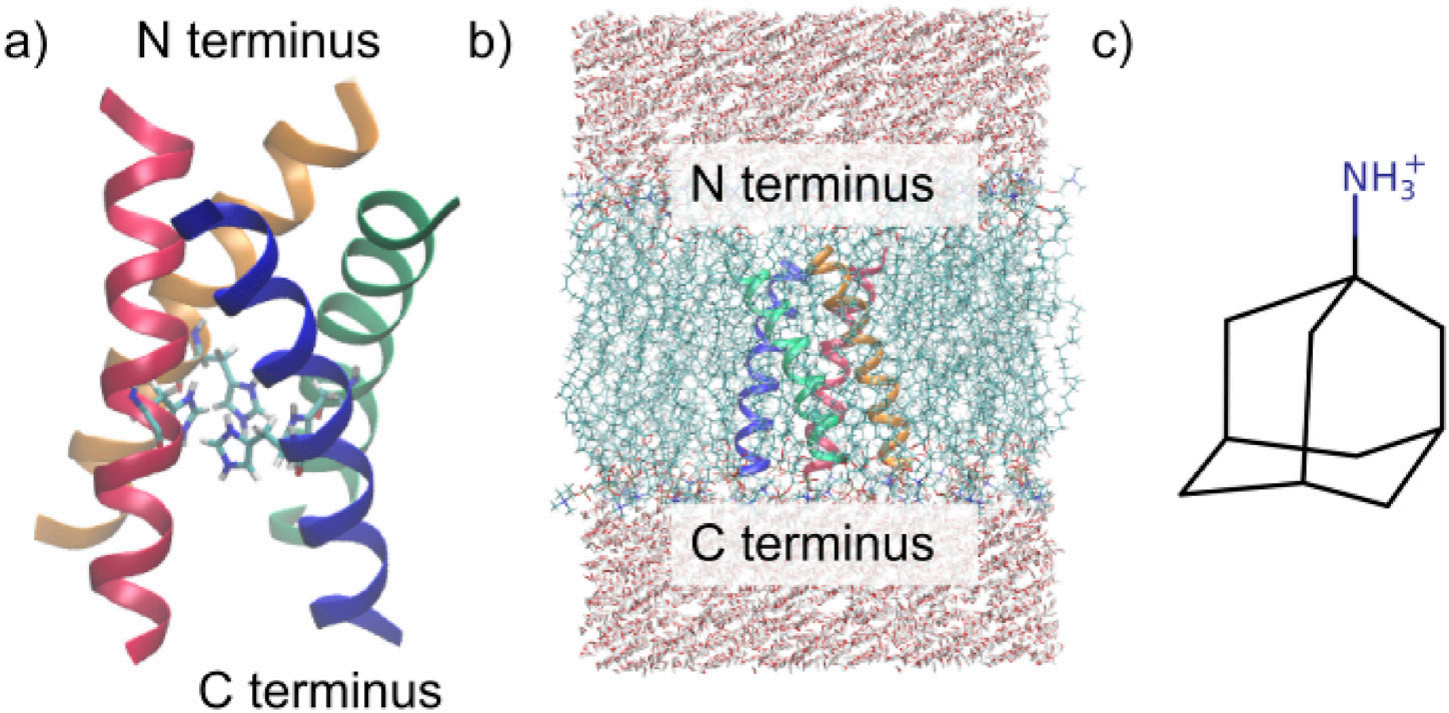
Investigated systems. **(a)** M2 channel in the 3LBW structure, including the four His37 residues. **(b)** Simulation box of the protein embedded in the POPC membrane surrounded by TIP3P water. **(c)** Structure of amantadine (Intharathep et al., 2008).

The membrane comprised ca. 60 POPC molecules per bilayer (Kass and Arkin, 2005). To accurately model the biological milieu, the membrane was embedded in a rectangular simulation box flanked by 22.5 Å water layers both above and beneath the membrane (roughly 3,500 water molecules altogether), achieving a balanced 1:1 ratio between the upper and lower membrane leaflets (see Figure 1b). ${\text{Na}}^{+}$ and ${\text{Cl}}^{-}$ ions were added to the aqueous solution at a concentration of 0.15 M to simulate physiological conditions. Additional anions were added to compensate for the protonation state of the protein. For the protein, three different PDB structures from the RCSB database were tested: 1NYJ is a high-resolution structure of the M2 protein in its closed state as derived from solid-state NMR spectroscopy (Nishimura et al., 2003), 2L0J depicts the complete structure of the M2 proton channel as reconstructed from solid-state NMR data (Sharma et al., 2010), and 3LBW offers a high-resolution (1.65 Å) crystallographic view of the M2 transmembrane domain (Acharya et al., 2010) (see Figure 1a), which was also used by Wang et al. (2011) in MD simulations investigating the effect of inhibitors. Among these structures, the transient RMSD of the last model demonstrated the greatest stability in non-polarizable simulations (refer to Supplementary Figure S1). Consequently, this structure was selected for all subsequent simulations as it most closely resembled the experimental structure. Furthermore, as shown in the Supplementary Figure S2, the RMSF of the amino acids in our trajectories correlate with the experimental B -factors of 3LBW. The simulations with the addition of amantadine (Figure 1c) were set up using the 6BKK structure, which is a high-resolution (2.00 Å) X-ray diffraction structure of the transmembrane domain bound to amantadine (Thomaston et al., 2018). Apart from the starting structure, the same workflow was used for both systems.

**FIGURE 2:**
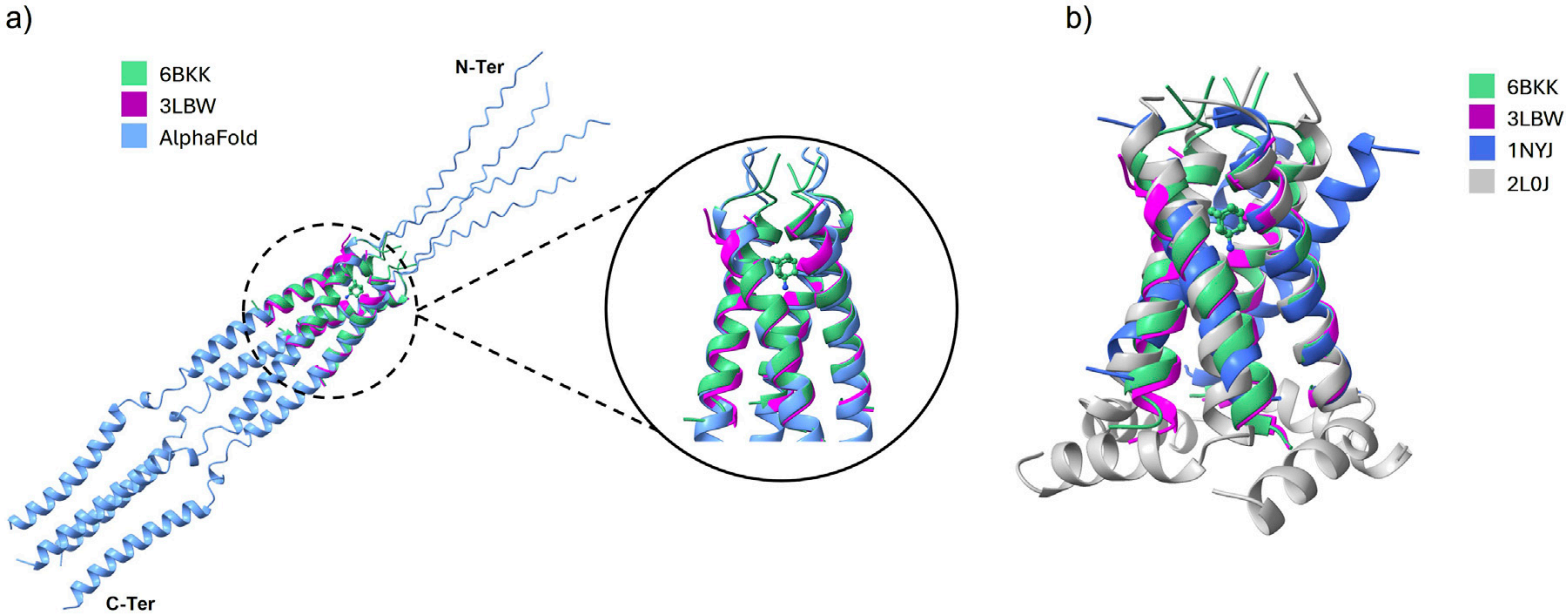
**(a)** The alignment of 3LBW (purple) and 6BKK (green) with the structure of the whole protein as suggested by AlphaFold (blue). The inset shows the binding position of amantadine in 6BKK. **(b)** Superimposed PDB structures of 3LBW (green), 6BKK (purple), 1NYJ (blue) and 2L0J (gray).

As shown in Figure 2a, the 3LBW and the 6BKK structures align very closely with the full structure suggested by AlphaFold 3 (Abramson et al., 2024). The superimposition in Figure 2b that 3LBW and 6BKK exhibit the closest alignment, whereas 1NYJ and 2L0J deviate from them (and thus from AlphaFold). These observations strongly support the use of 3LBW and 6BKK for further analysis, as their high degree of similarity facilitates direct comparison of simulations with and without amantadine.

### 2.2 Trajectory production

The gating of the channel and the binding of amantadine both take place entirely in the transmembrane part of the protein (Duff and Ashley, 1992; Wang et al., 1995). Thus, we focused our investigations on this specific region of the protein, as taking a much larger structure would make the already quite resource-intensive simulations even more costly and the overall goal of this study is the comparison of polarizable and non-polarizable force fields for the protein and water.

After setting up the simulation box, CHARMM (Brooks et al., 2009) was applied to create a minimized starting structure and to make the atoms polarizable (if necessary) using Drude particles (Lamoureux et al., 2006; Baker et al., 2010; Lopes et al., 2013; Lemkul et al., 2016; Kumar et al., 2019; Lin et al., 2020). We applied the CHARMM36m force field (Huang et al., 2016) for the membrane and protein. Consequently, the water models TIP3P (Jorgensen et al., 1983; Mark and Nilsson, 2001) and SWM4 (Lamoureux et al., 2003; Sega and Schröder, 2015) were employed for the non-polarizable and polarizable water, respectively.

All MD simulations were performed in OpenMM 7.6 (Eastman et al., 2017) using a Velocity Verlet integrator (Gong and Padua, 2021). In the case of polarizable molecules, the mass of the mobile Drude particle was set to 0.4 au, and the vibrations of the Drude oscillator were kept at a temperature of 1 K. The maximum allowed distance between a Drude particle and its parent atom was 0.2 Å, the Drude force constant was 1,000 kcal mol^−1^ Å^−2^. The Particle Mesh Ewald method (Darden et al., 1993; Essmann et al., 1995), with an error tolerance of 0.0005, was selected for the electrostatic cutoff (Darden et al., 1993; Essmann et al., 1995), while van der Waals interactions were managed using the Force-switch method, applying a switch-on distance of 1.0 nm and a switch-off distance of 1.2 nm. Positional restraint force constants were applied to protein backbones, side chains, lipids, and dihedral restraint force constants for lipids, adhering to the default parameters set by CHARMM-GUI.

The simulation protocol was conducted under the NpT ensemble conditions, maintaining a temperature of 303.15 K and a pressure of 1 atm. Following a comprehensive system equilibration of 5 ns using CHARMM-GUI’s default settings, the production phase proceeded over an extensive duration of 100 ns, with a time step of 2 fs for the completely non-polarizable systems and 1 fs for the (partially) polarizable ones. This led to a total simulation period of 14.4 µs of (partially) polarizable systems, which are generally about four times as expensive as non-polarizable ones (Antila et al., 2024).

### 2.3 Analysis techniques

Our analysis of the trajectories is based on the Python-based library MDAnalysis (Michaud-Agrawal et al., 2011; Gowers et al., 2016), which offers standard routines for calculating the common transient RMSD values and diffusion coefficients. All RMSD values were calculated on the backbone atoms of the protein, with the first frame of the production run (after equilibration) as the reference. Using our extension (Github, 2024), the spatial structure can be analyzed in terms of the radial distribution function $g_{ij}^{000}\left(r\right)$ and the orientational correlation function $g_{ij}^{011}\left(r\right)$ , which characterizes (see Equation 1) the orientation of the dipole $\mu_{j}$ of molecule j (see Equation 2) with respect to the position of reference site i (Blum and Nartan, 1976; Steinhauser and Bertagnolli, 1981; Schröder et al., 2007).

$$g_{ij}^{000}\left(r\right)=\frac{1}{4\pi r^{2}dr\rho }\underset{j}{\sum }\delta \left(r-r_{ij}\right)$$ (1)

$$g_{ij}^{011}\left(r\right)=\frac{1}{4\pi r^{2}dr\rho }\underset{j}{\sum }\cos\left({\vec{r}}_{ij},{\vec{\mu }}_{j}\right)\cdot \delta \left(r-r_{ij}\right)$$ (2)

**FIGURE 3:**
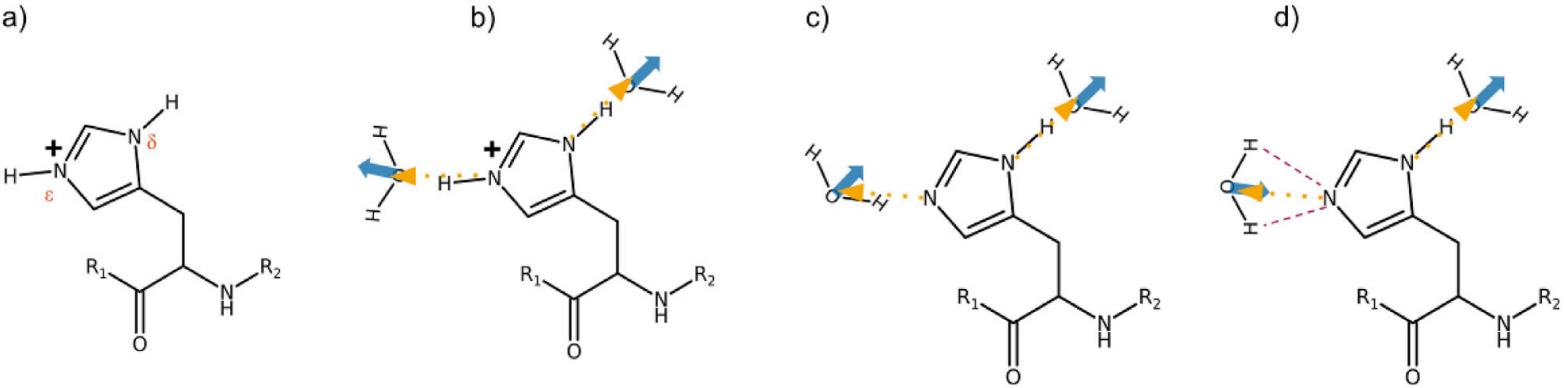
**(a)** Nomenclature of the histidine nitrogens. **(b)** $g^{011}\left(r\right)$ in the case of the protonated histidine, HSP. The dashed yellow arrow is $r_{ij}$ pointing to the center of mass of the water. The blue arrow is the dipole moment of water. Since both point in the same direction, the cosine $\cos\left({\vec{r}}_{ij},{\vec{\mu }}_{j}\right)$ is close to 1. **(c)** Neutral histidine HSD with two water molecules. The single hydrogen bond (HB) to the ${\text{N}}^{\epsilon }$ is characterized by a cosine close to zero, and consequently, $g^{011}\left(r\right)$ is small. **(d)** Neutral histidine HSD with a bifurcated hydrogen bonding at ${\text{N}}^{\epsilon }$ (red dashed lines). Since the cosine $\cos\left({\vec{r}}_{ij},{\vec{\mu }}_{j}\right)$ is almost −1, the corresponding $g^{011}\left(r\right)$ will be negative.

A co-linear arrangement of the hydrogen bond of the protonated ${\text{N}}^{\epsilon }$ of histidine with water results in large values for the cosine $\cos\left({\vec{r}}_{ij},{\vec{\mu }}_{j}\right)$ and consequently large values for $g_{ij}^{011}\left(r\right)$ (see Figure 3b). In contrast, if both water hydrogen atoms point towards the histidine ${\text{N}}^{\epsilon }$ , the $\cos\left({\vec{r}}_{ij},{\vec{\mu }}_{j}\right)$ becomes significantly negative (Figure 3d). If only one of the water hydrogen points towards the nitrogen, the angle is roughly 90° and hence $g_{ij}^{011}\left(r\right)$ diminishes (see Figure 3c). Of course, the distance behavior of $g_{ij}^{000}\left(r\right)$ and $g_{ij}^{011}\left(r\right)$ already tells about the strength of the hydrogen bond: the shorter the distance at the first peak maxima, the stronger the hydrogen bond.

**FIGURE 4:**
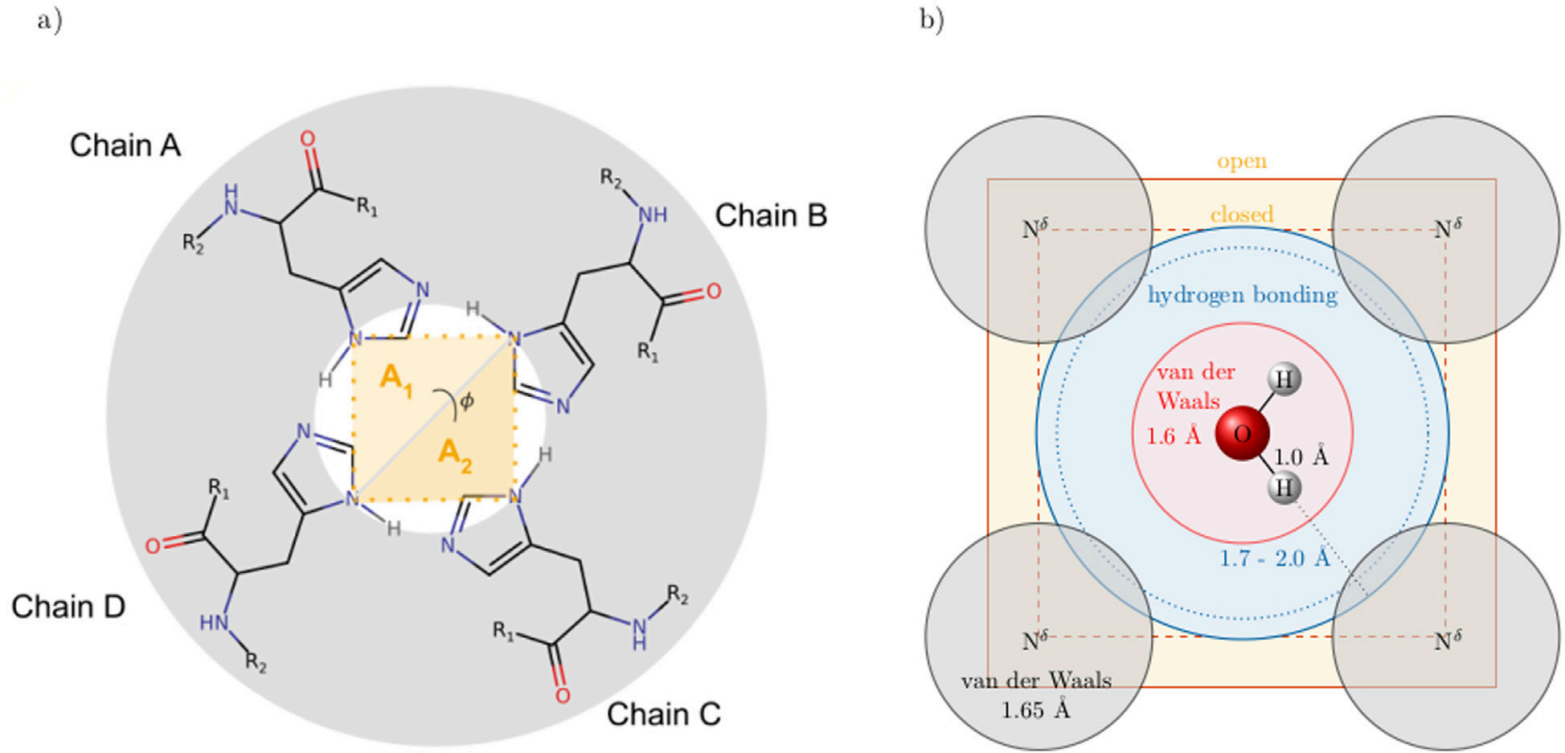
**(a)** Cross-section of the ion channel at the height of the His37 tetrad. The opening and closing of the channel can be characterized by an area $A=A_{1}+A_{2}$ spanned by the four ${\text{N}}^{\delta }$ of the histidines. The angle ϕ detects if the helices of the chains start to bend. **(b)** A water molecule crossing this area. The red and gray spheres are the van-der-Waals spheres of the oxygen and nitrogens, respectively. The blue area indicates that all atoms within may hydrogen bond to the hydrogens.

The channel opening and closing may be characterized by an area between the ${\text{N}}^{\delta }$ of the His37 residues of the four chains A, B, C, and D of the ion channel as illustrated in Figure 4a. Even if the nitrogens are not on the same plane, the calculation via the two triangles ensures easy computation. The vectors ${\vec{n}}_{1}=r_{N^{\delta }N^{\delta }}\left(BD\right)\times r_{N^{\delta }N^{\delta }}\left(BA\right)$ and ${\vec{n}}_{2}=r_{N^{\delta }N^{\delta }}\left(BD\right)\times r_{N^{\delta }N^{\delta }}\left(BC\right)$ are perpendicular to the triangle areas $A_{1}$ and $A_{2}$ , respectively. Consequently, the areas are $A_{1}=\frac{1}{2}\left|{\vec{n}}_{1}\right|$ and $A_{2}=\frac{1}{2}\left|{\vec{n}}_{2}\right|$ , and the angle ϕ between these two triangles $\cos\left(\phi \right)=\frac{{\vec{n}}_{1}\cdot {\vec{n}}_{2}}{\left|{\vec{n}}_{1}|\cdot |{\vec{n}}_{2}\right|}$ gives information on the bending of the channel.

## 3 Results

### 3.1 At the level of the ion channel

**FIGURE 5:**
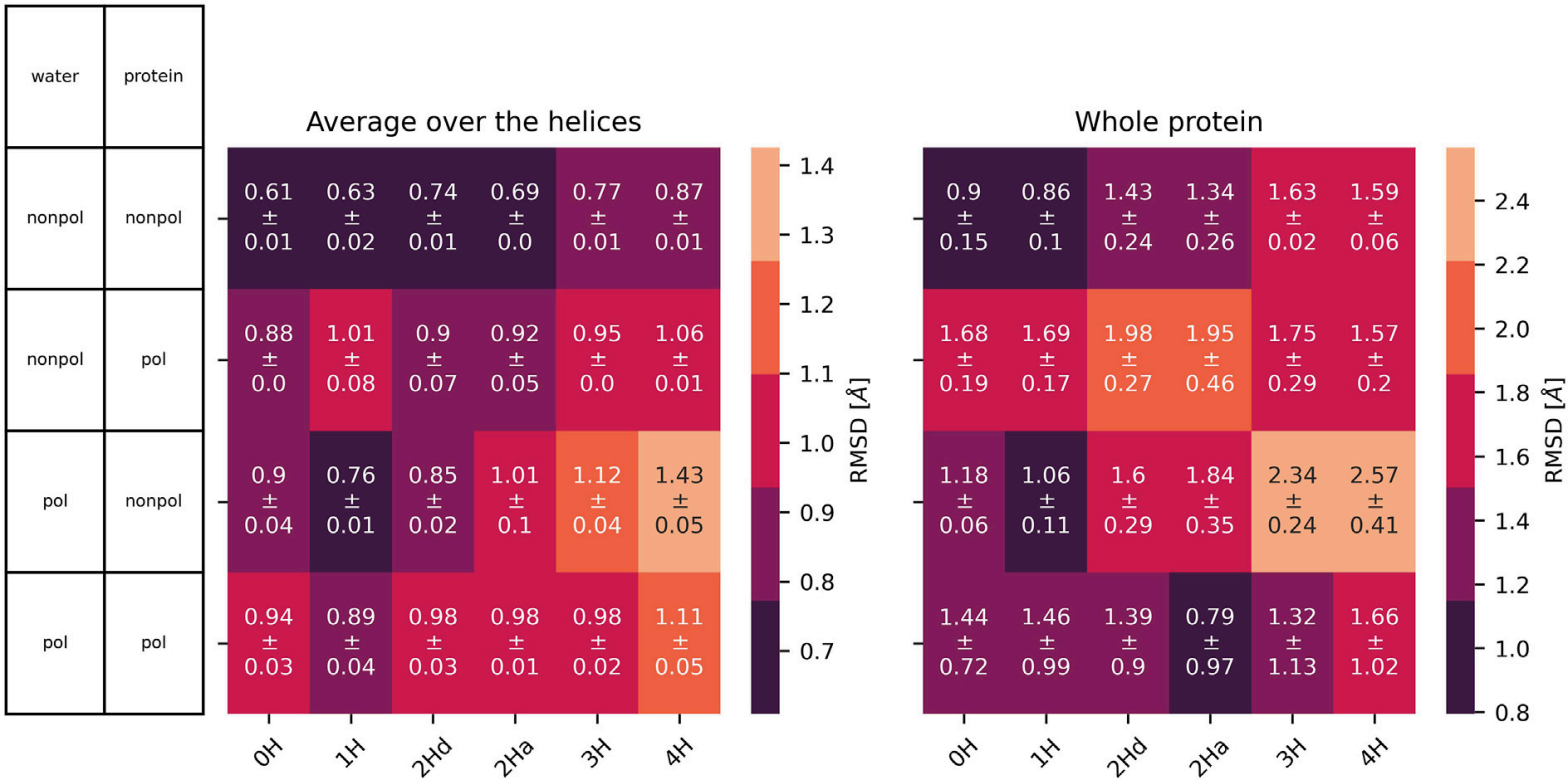
RMSD with standard deviation between the three replicas of the embedded protein averaged over all four chains (left) and of the whole protein (right) as a function of the protonation state and polarizability. In 2Hd and 2Ha, the diagonal and adjacent histidines are protonated, respectively.

All protonation states and polarizabilities resulted in stable simulations with highly conserved protein structures, which are reflected in the low root mean square deviations RMSD in the heat map of Figure 5. The consistently low RMSD values can be attributed to the ion channel’s embedding in a membrane, which limits significant deformation of the secondary structure. The RMSD(t) was computed individually for each of the four chains. Subsequently, the corresponding average was modeled by a bi-exponential decay model in Equation 3

$$RMSD\left(t\right)=\overset{2}{\underset{k=1}{\sum }}A_{k}\left(1-\exp\left(-t/\tau_{k}\right)\right)$$ (3)

where the sum of the amplitudes $A_{k}$ corresponds to the equilibrated RMSD -values presented in the heat map. These are a function of the force field combination and the protonation state. In all force field combinations, an incremental increase in RMSD values correlates with higher protonation states of the M2 channel. Up to a protonation state of +2, non-polarizable protein systems exhibit lower RMSD -values than polarizable protein systems. When three or four histidines are protonated, the fully non-polarizable system (non-polarizable water and non-polarizable protein) continues to display the lowest RMSD -values, while the non-polarizable protein interacting with polarizable water exhibits the highest RMSD -values. The water model’s polarizability appears to have a minor effect on the stability of the polarizable protein, as evidenced by the comparable RMSD -values shown in Figure 5.

**FIGURE 6:**
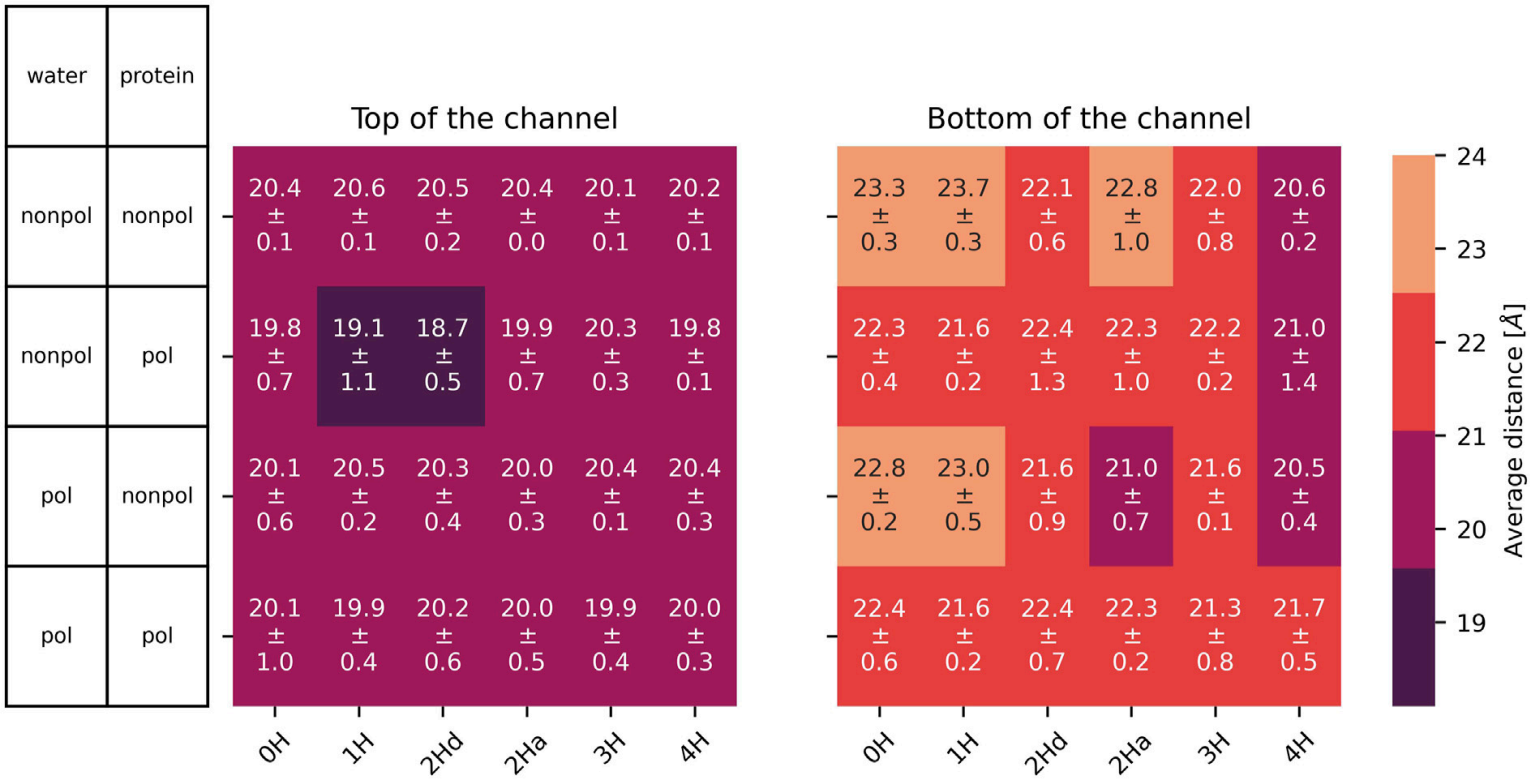
Average distance with standard deviation between opposite ${\text{C}}^{\alpha }$ at the top (left) and bottom (right) of the channel. In 2Hd and 2Ha, the diagonal and adjacent histidines are protonated, respectively.

In addition to the RMSD , the diagonal distance of the ${\text{C}}^{\alpha }$ at the top and bottom of the channel in Figure 6 also characterizes the state of the ion channel. At the top of the M2 channel, the cross distances remain consistently around 20 Å, irrespective of the polarizability of the water, the polarizability of the protein, or the protonation state of the four histidines at the middle of the ion channel. These cross-distances agree with a distance of 13.2 Å between adjacent helices, reported by Sansom et al. (1997). The cross-distances at the bottom of the ion channel are larger by two or more Å. Moreover, the behavior of systems with polarizable proteins exhibits greater similarity to one another compared to those with non-polarizable proteins, which also show internal consistency. In contrast, the polarizability of the water appears to have a minimal effect on these distances. The cross distances at the bottom and top of the channel are sufficiently large to allow multiple water molecules to enter or exit the channel simultaneously. Interestingly, they tend to decrease with increasing protonation of the histidines, despite the spatial separation between the histidine ring and the bottom of the channel (see Figure 1). This phenomenon may be attributed to the rigidity of the four helices that comprise the channel, where the expansion of the cross-distances at the histidine tetrad, driven by higher protonation states, induces a compensatory reduction in the distances at the bottom of the channel.

**FIGURE 7:**
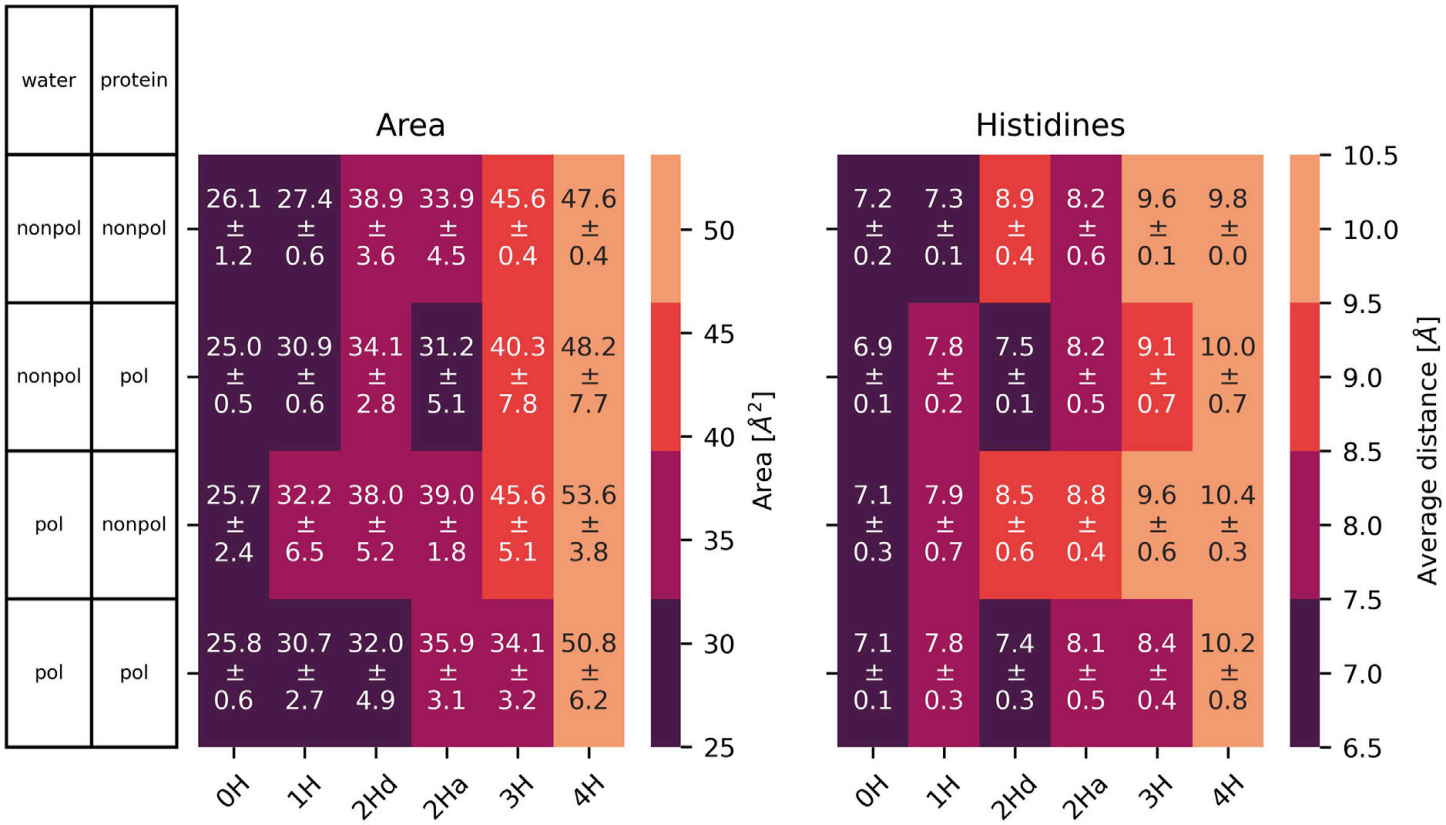
Left: area spanned by the ${\text{N}}^{\delta }$ of the His37 with standard deviation (see Figure 4a for details). Right: average distance and standard deviation between the ${\text{N}}^{\delta }$ of opposite His37. In 2Hd and 2Ha, the diagonal and adjacent histidines are protonated, respectively.

The radius of the channel throughout the membrane was analysed with HOLE (Smart et al., 1993, Smart et al., 1996). The program seems unable to handle cases where the channel is split between primary and image cells, thus some of the results were unsatisfactory. Still, the resulting radii are shown in the supplementary material (Supplementary Figures S5–S7 for the simulations without the inhibitor and Supplementary Figures S18–20 with inhibitor). The results mainly agree with the distances shown in Figures 6, 7. We focus on these single-value distances for an easier comparison between systems.

### 3.2 At the bottleneck

The cross distances between the ${\text{N}}^{\delta }$ atoms of His37 residues in the middle of the M2 channel are illustrated in Figure 7. These distances correspond to the diameter of the pores in Chen et al. (2016) at a pH of 6. They are significantly smaller than the ${\text{C}}^{\alpha }$ cross distances in Figure 6 as the ${\text{N}}^{\delta }$ s are part of the histidine side chains pointing inwards the channel. Moreover, the histidines appear to form a bottleneck within the channel. As expected, the cross-distances increase with higher protonation states due to the charged histidines’ Coulombic repulsion. This trend is consistently observed across all (non-)polarizable force field combinations. Figure 7 also shows the area spanned by the four histidine ${\text{N}}^{\delta }$ atoms as explained in Figure 4. These areas follow a similar trend to the cross distances. In the closed state of the channel at low protonation states, an area of roughly 26 Å^2^ is observed, while in the open case, this area nearly doubles. In Figure 4b, the relevant spatial constraints are illustrated by the solid (open channel) and dashed (closed channel) yellow lines. The Lennard-Jones radius of the histidine nitrogen atoms located at the channel corners is 1.65 Å and represented by the gray spheres, while the water oxygen exhibits a slightly smaller Lennard-Jones radius of 1.6 Å (red sphere). Based purely on steric considerations, the channel appears sufficiently wide to permit water passage as also argued by previous studies on aquaporins (Verkman and Mitra, 2000); Zhang et al., 1993) employing a hydrodynamic model. They reported an effective pore radius of approximately 1.9 Å for the water-conducting channel, which would be sufficient for the water molecule to pass the bottleneck in our study. However, considering hydrogen-bonding of the water indicated by the blue sphere and its overlap with the gray spheres, traversal through the closed bottleneck is likely hindered by energetic constraints. Typical hydrogen bond lengths range from 1.7 Å to 2.0 Å (Harris and Mildvan, 1999). Even in cases where the area reaches 55 Å^2^ (solid yellow line), we expect that only a single water molecule can traverse the histidine ring at a time within the M2 channel (see Figure 4b).

**FIGURE 8:**
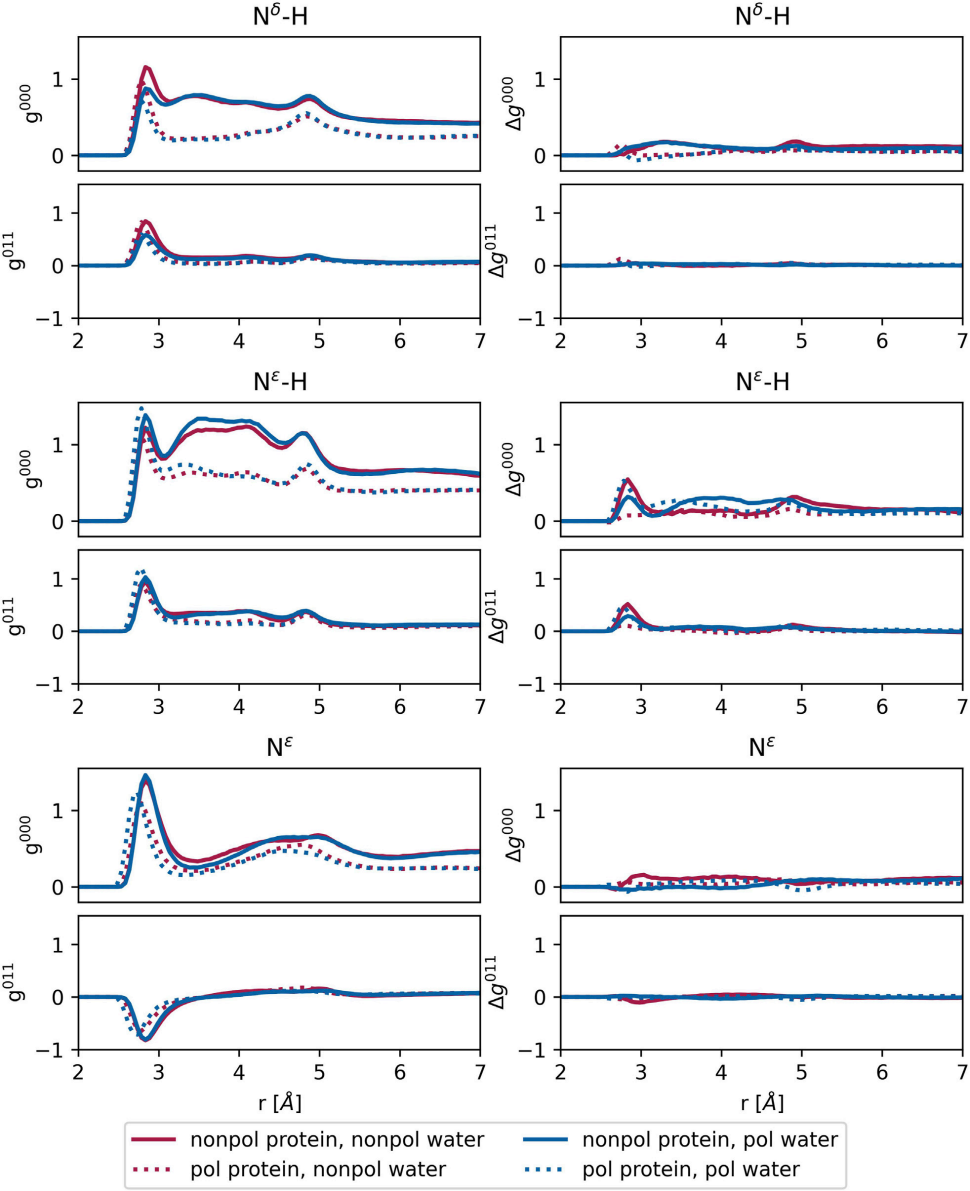
Left: radial distribution functions $g^{000}$ and $g^{011}$ for each N of the histidines described in Figure 3 and water. Right: difference between the radial distribution functions $g^{000}$ and $g^{011}$ with and without amantadine. Averages over all protonation states and replicas.

The small volume of the channel is further evidenced by the long-distance limit of $g^{000}\left(r\right)$ in Figure 8, which is significantly lower than unity. At first glance, the first peak of $g^{000}\left(r\right)$ appears at approximately 2.8 Å, with a second peak at 4.9 Å for both protonation states of nitrogen, ${\text{N}}^{\delta }$ -H and ${\text{N}}^{\epsilon }$ -H. The region between these peaks is not characterized by a distinct minimum but rather by a prominent plateau, which lies above the average value. This plateau is less pronounced for the deprotonated ${\text{N}}^{\epsilon }$ . In addition, the first $g^{000}\left(r\right)$ peak is higher in this case. Closer inspections reveal that the first peak in the polarizable protein systems is located at 2.785 Å independent of the water polarizability. In contrast, this distance is increased by 0.05 Å in non-polarizable protein systems, a trend that also holds for deprotonated ${\text{N}}^{\epsilon }$ . The second peak appears at 4.835 Å in polarizable protein systems, while in non-polarizable systems, this distance is reduced by 0.05–0.1 Å.

For protonated histidine nitrogen, the $g^{011}\left(r\right)$ functions exhibit positive peaks at the first coordination shell, indicating hydrogen bonding with water molecules, consistent with the behavior shown in Figure 3b. This trend occurs regardless of the polarizability of the protein or the water molecules. In contrast, the $g^{011}\left(r\right)$ for deprotonated ${\text{N}}^{\epsilon }$ show negative peaks, suggesting a preference for bifurcated hydrogen bonding, as depicted in Figure 3d. Single hydrogen bonding, as illustrated in Figure 3c, appears less likely in this case.

## 4 Discussion

### 4.1 Effect of the polarizability

Polarizable water models are essential for the future development of proton transfer models for water and histidines. The proton transfer within the channel may not only occur between water molecules in a Grotthus mechanism style but may also involve the channel’s histidine, as their pKa is close to physiological conditions. Accordingly, our focus extends to investigating the changes that arise when transitioning from non-polarizable to polarizable water models and their impact on the surrounding membrane and protein structures. In summary, both methods demonstrated comparable protein stability, as indicated by the root mean square deviations shown in Figure 5. Differentiating the treatment of system components (e.g., polarizable water with non-polarizable protein) did not present any issues. This finding is significant for maintaining low computational costs in future applications of Protex (Joerg et al., 2023; Gődény et al., 2024), which will handle the proton transfers in classical MD simulations. Generally, polarizable simulations are at least four times more computationally expensive than non-polarizable ones. We believe that a substantial portion of these costs can be mitigated by restricting the polarizable treatment to only those regions of the system that are involved in proton transfer.

Overall, the water polarizability appears to have minimal influence on the RMSD , cross-distances, or the area of the histidine tetrad. However, in the case of the radial distribution functions for protonated nitrogen, as shown in Figure 8, the height of the first peak is affected by the water’s polarizability. Specifically, for ${\text{N}}^{\delta }$ -H, the peak is significantly higher with non-polarizable TIP3 water compared to polarizable SWM4 water, while for ${\text{N}}^{\epsilon }$ -H, the opposite trend is observed. The polarizability of the protein has a more pronounced effect, which is especially noticeable in the $g^{000}\left(r\right)$ of the protonated nitrogen atoms, in the region above 3 Å, which corresponds to the second water shell around the histidines. The two solid lines in the top and middle panel of Figure 8 look very similar to each other, as do the two dashed ones, meaning that switching on polarizability for the protein makes a larger difference than treating water in a polarizable manner. As induced dipoles usually counteract Coulombic interactions, the attraction of water to the protonated histidines in the second shell is reduced resulting in lower coordination numbers in the second shell. The first shell is less affected since the direct hydrogen bonds between the water oxygen and the histidine hydrogens are strong.

The RMSD is slightly higher for polarizable proteins, and the cross-distances are smaller at low protonation states. While the cross-distances and area of the histidine tetrad do not directly depend on protein polarizability, a slight shift to shorter distances in the first peak of the radial distribution function for histidine nitrogens and water is observed. The region between the first and second peaks is also more prominent in non-polarizable protein systems.

In summary, the protein’s polarizability induces some structural changes in the channel. However, key characteristics at the bottleneck, such as the area of the His37 tetrad and cross-distances, remain unaffected. Therefore, if our future polarizable simulations reveal discrepancies compared to behaviors observed in non-polarizable simulations from the literature, these differences are likely attributable to the enabled proton transfer rather than the polarizable forces themselves.

### 4.2 Effect of amantadine

**FIGURE 9:**
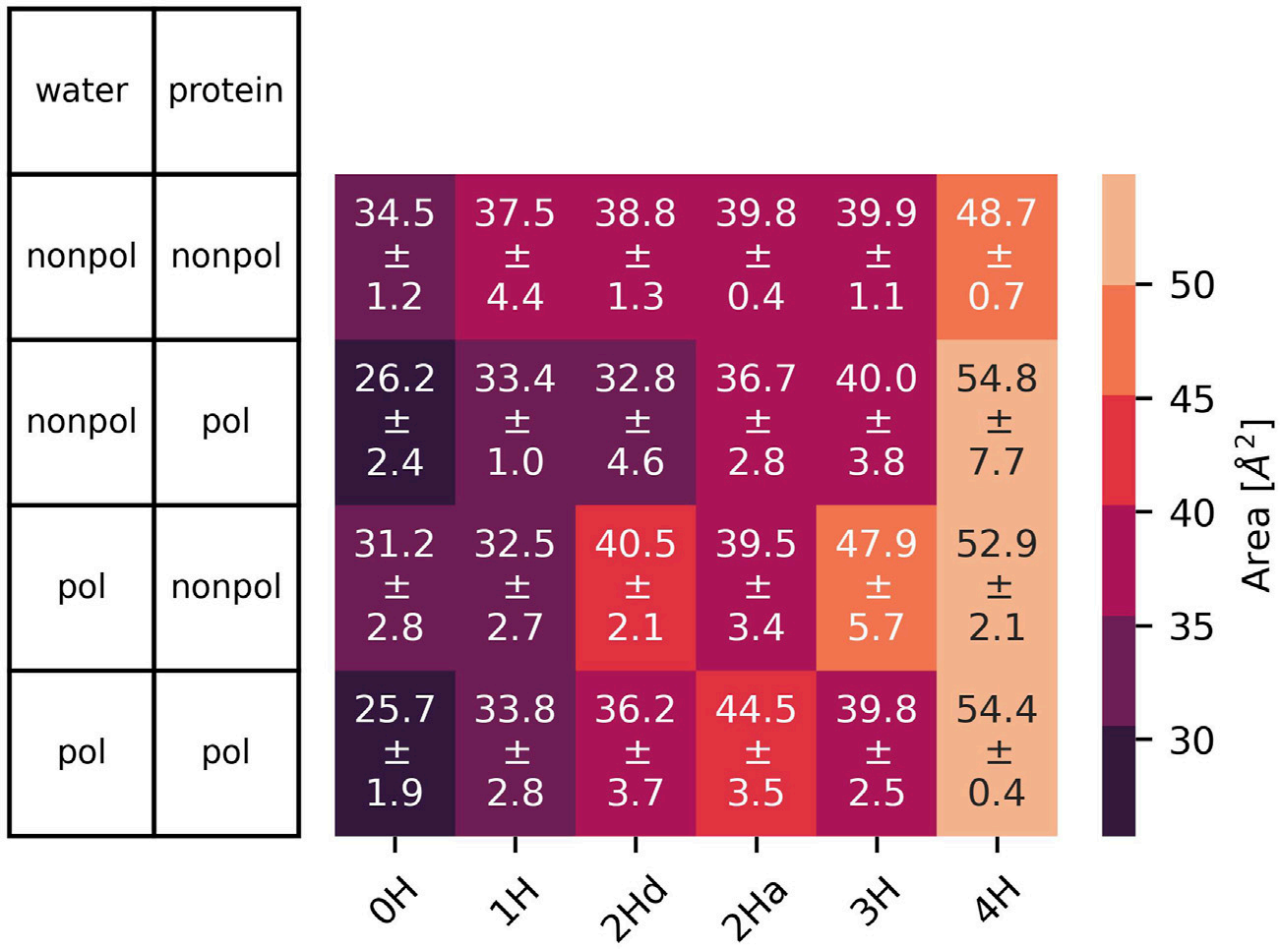
Area spanned by the ${\text{N}}^{\delta }$ of the His37 and standard deviation between the three replicas in the simulations with amantadine (see Figure 4 for details). In 2Hd and 2Ha, the diagonal and adjacent histidines are protonated, respectively.

The simulations that include amantadine (see Figure 1) exhibit similar trends to those without the ligand. On average, the channel diameter is slightly larger at the top, middle, and bottom of the channel compared to the ligand-free simulations, with a small deviation ( ± 0.5 Å at the bottleneck and ± 1.5 Å at the chain ends, relative to Figure 6). This variation could be attributed to the different crystal structures used for simulation setup or simply due to the ligand requiring slightly more space. The increased flexibility at the chain ends may also contribute to the larger deviation. We (Intharathep et al., 2008) already reported that the amantadine has no significant interactions with the channel due to its allosteric hindrance. Nonetheless, the overall trends remain consistent: the protonation state does not affect the diameter at the top or bottom of the channel, while the distance between opposing His37 residues increases with higher protonation states. The same holds for the area spanned by the ${\text{N}}^{\delta }$ atoms (Figure 9): the area is generally slightly larger in the presence of the ligand, but the increase with protonation state persists.

The impact of amantadine on the radial distribution functions between the histidine nitrogens at the bottleneck and water is minimal, as evidenced by the difference plots $\Delta g^{000}\left(r\right)=g^{000}\left(r,\text{amantadine}\right)-g^{000}\left(r\right)$ shown in Figure 8. There is virtually no noticeable effect for both protonated ${\text{N}}^{\delta }$ and deprotonated ${\text{N}}^{\epsilon }$ . Only in the case of protonated ${\text{N}}^{\epsilon }$ -H, a significant increase in the first peak is observed. However, this does not correlate with the tetrad area, as the calculation of the area is based on the ${\text{N}}^{\delta }$ atoms.

**FIGURE 10:**
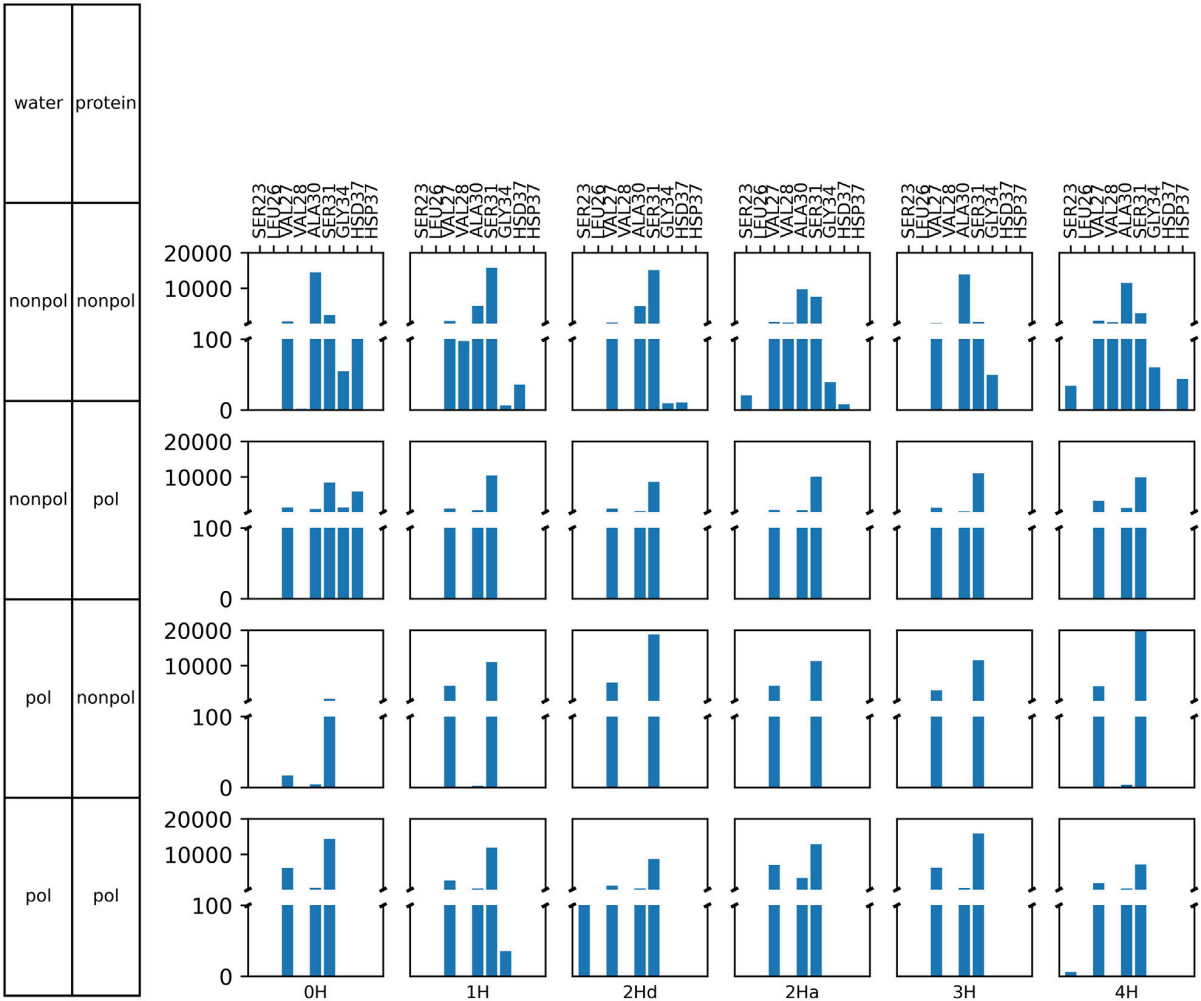
Frequency of hydrogen bonds between the amino hydrogens of amantadine and various amino acids of M2.

All this information suggests that the ligand does not fundamentally alter the channel structure or its response to protonation. Instead, amantadine appears to block the channel mechanically, leaving no space for water molecules to pass through. This is also supported by the analysis of the hydrogen bonds shown in Figure 10. We defined a hydrogen bond with a distance of maximum 4 Å between the donor and acceptor heavy atoms and a donor-H-acceptor angle of at least 120°. In agreement with previous results (Intharathep et al., 2008), amantadine interacts in all cases with Ala30 or Ser31 the most, thus forming a blockage close to the opening of the channel. Interaction with the His37 tetrad was very rarely observed. Visual inspection shows that amantadine stays very close to its starting position, and mainly just rotates in place. This is also confirmed by the very small diffusion coefficients (Supplementary Figure S11), as well as by the overwhelmingly large number of contacts to Ala30 or Ser31, as opposed to other residues. This is the case both for hydrophobic contacts (Supplementary Figures S12–S15) and hydrogen bonds (Figure 10).

## 5 Conclusion

Our simulations reveal that while polarizability induces subtle changes in the channel’s structure, key features at the bottleneck, such as the cross-distances and the area spanned by the histidine residues, remain largely unaffected. This suggests that any deviations observed in the future between polarizable and non-polarizable models are likely due to proton transfer processes rather than the polarizable forces themselves. Moreover, including amantadine as a ligand did not drastically alter the channel’s overall behavior, supporting the hypothesis that amantadine mechanically blocks the channel without inducing significant structural changes. These findings provide a foundation for further investigation into the role of proton transfer in the channel’s operation. The results also offer valuable insights for future computational studies, highlighting the potential for reducing computational costs by selectively applying polarizable models only to regions of interest, such as those involved in proton transfer.

## Data Availability

The raw data supporting the conclusions of this article will be made available by the authors, without undue reservation.
