# Supplementary material for: The interplay of polarizable water and protein in the activation of the M2 channel
Source: Front Pharmacol. 2025 Sep 2;16:1532697. doi: 10.3389/fphar.2025.1532697 (PMC12436341; doi:10.3389/fphar.2025.1532697)
Supplement: Supplementary file 1 [file Supplementaryfile1.pdf]

# Supplementary Material

## 1 METHODS

In the present study, we conducted molecular dynamics (MD) simulations of the M2 channel protein embedded in a POPC membrane. Since our objective was to modify the protonation state of the histidine tetrad and to evaluate the effects of both non-polarizable and polarizable force fields on the membrane, protein, and surrounding water, it was imperative to ensure that the simulated protein maintained its structural integrity and remained consistent with the experimental structure. To achieve this, we initiated $500\,\mathrm{ns}$ simulations using the PDB structures 3LBW (Acharya et al., 2010), 1NYJ (Nishimura et al., 2003), and 2L0J (Sharma et al., 2010).

### 1.1 Root-Mean-Squared-Displacement

The root-mean-squared displacement (RMSD) quantifies deviations between the simulated structure and a reference structure. In this case, the reference corresponds to the first frame of the trajectory.

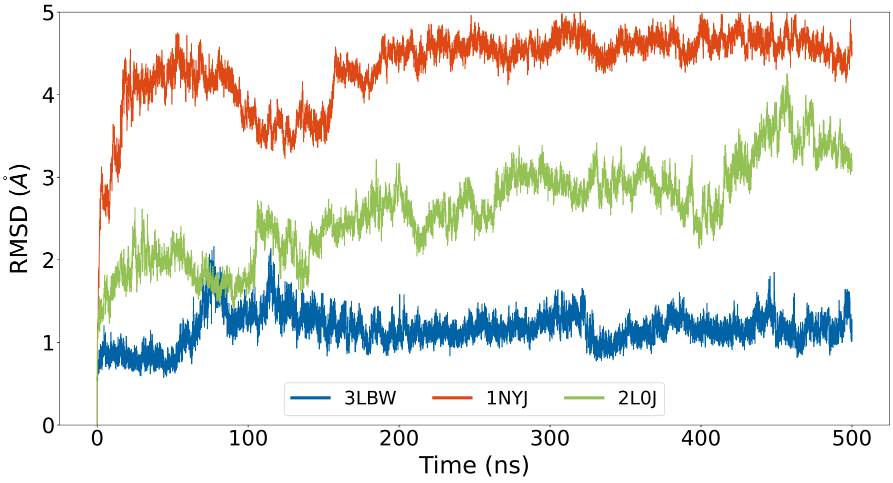


**Figure S1.** RMSD of the entire protein in simulations initiated from three different PDB structures.

The RMSD values of the investigated protein structures are presented in Fig. S1. As expected, the protein undergoes structural equilibration during the MD simulations. Consequently, the RMSD increases initially before stabilizing at a later stage. However, the equilibration behavior varies among the three examined structures: The 1NYJ structure equilibrates rapidly within the first $50\,\mathrm{ns}$, but it exhibits a relatively high RMSD value of approximately $4.5\,\text{Å}$. In contrast, 2L0J does not reach equilibration within the entire simulation time and continues to show increasing RMSD values beyond $500\,\mathrm{ns}$, rendering it unsuitable for further investigations. In comparison, the 3LBW structure achieves equilibrium after approximately $100\,\mathrm{ns}$, with a low RMSD value of $1.3\,\text{Å}$. This low RMSD suggests that the simulated structure remains closely aligned with the experimental conformation throughout the simulation period. Consequently, 3LBW was selected as the starting structure for all subsequent investigations.

## 1.2 Root-Mean-Squared-Fluctuations

The RMSD was calculated for the entire protein, whereas the root-mean-squared fluctuations (RMSF) were computed for each residue individually. To further analyze protein flexibility, we restarted the trajectory production of the 3LBW structure at protonation state 0 and conducted simulations using three independent replicas. For each replica, the RMSF was determined for all four protein chains.

According to Kuzmanic and Zagrovic (2010), the square of the RMSF is correlated with the experimentally derived B-factors:

$$RMSF_i^2 \propto B_i \tag{S1}$$

Since the B-factors provided in the PDB dataset are reported at atomic resolution, we averaged them over each amino acid residue to obtain $B_i$. Additionally, the values were scaled to align with the magnitude of the computational RMSF values for direct comparison.

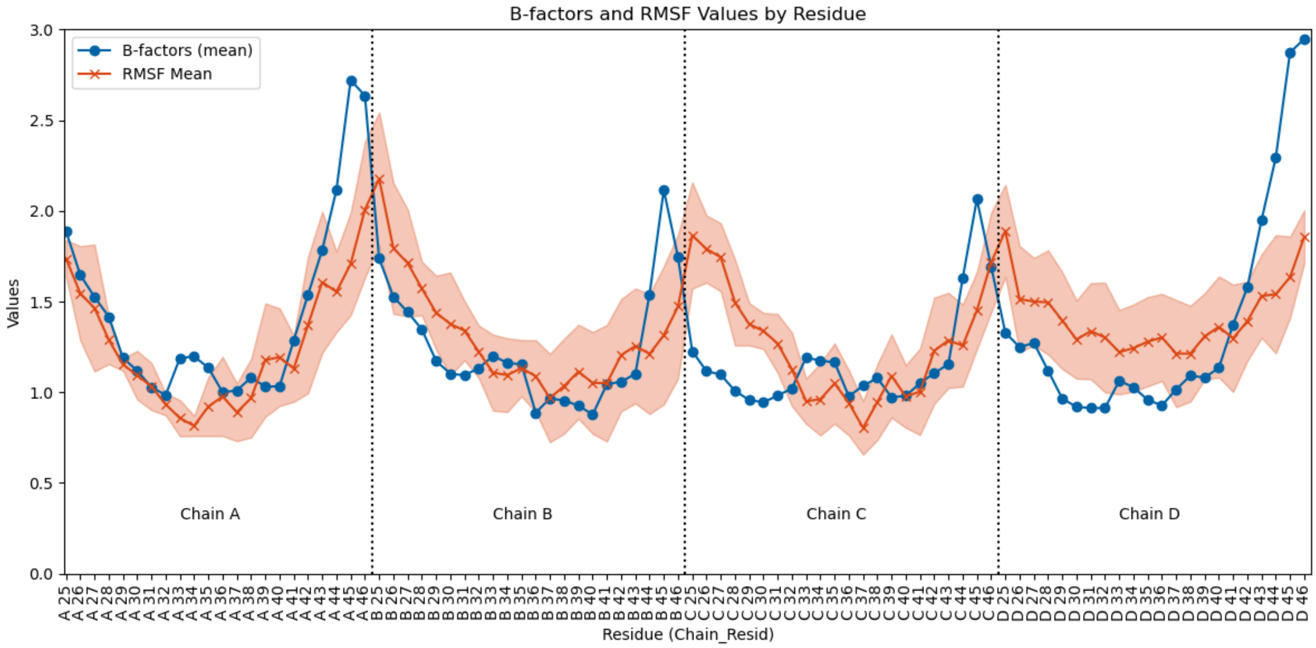


**Figure S2.** Correlation between computational $RMSF_i^2$ and experimental B-factors.

The RMSF for each residue is highly sensitive to structural fluctuations. Therefore, to ensure robustness, we computed RMSF values across all replicas and over the full simulation period. The results of the correlation described in Eq.(S1) are presented in Fig.S2. The large orange regions in Fig. S2 represent the standard deviation of $RMSF_i$. It should be noted that the presented comparison corresponds to the simulation using a polarizable membrane/protein and polarizable water force field. However, other force field combinations produced comparable results. Overall, a strong correlation was observed between the computational RMSF and the experimental B-factors, with only minor deviations detected in the N-terminal region of chain C and the C-terminal region of chain D. Based on these findings, we consider the 3LBW structure (Acharya et al., 2010) to be suitable for our analysis.

# 2 RESULTS

The histidine tetrad in our protein can adopt six distinct protonation states: 0, +1, +2 (adjacent), +2 (diagonal), +3, and +4. Additionally, we examined four different force field combinations: Polarizable protein + polarizable water, polarizable protein + non-polarizable water, non-polarizable protein + polarizable water, and non-polarizable protein + non-polarizable water. To enhance statistical reliability, MD simulations were conducted using three independent replicas, each running for 100 ns.

## 2.1 Root-Mean-Squared-Displacement

The RMSD of the whole channel is shown in Fig. S3, calculated only for the backbone atoms and Fig. S4, calculated for all atoms of the protein . As expected, the backbone shows a lower RMSD in all cases. The RMSD of the backbone is in all cases very low and stable. Most systems with amantadine show a slightly lower RMSD with nonpolarizable protein, however, the difference is small, and the trend is not consistent over the various protonation states and force field combinations.

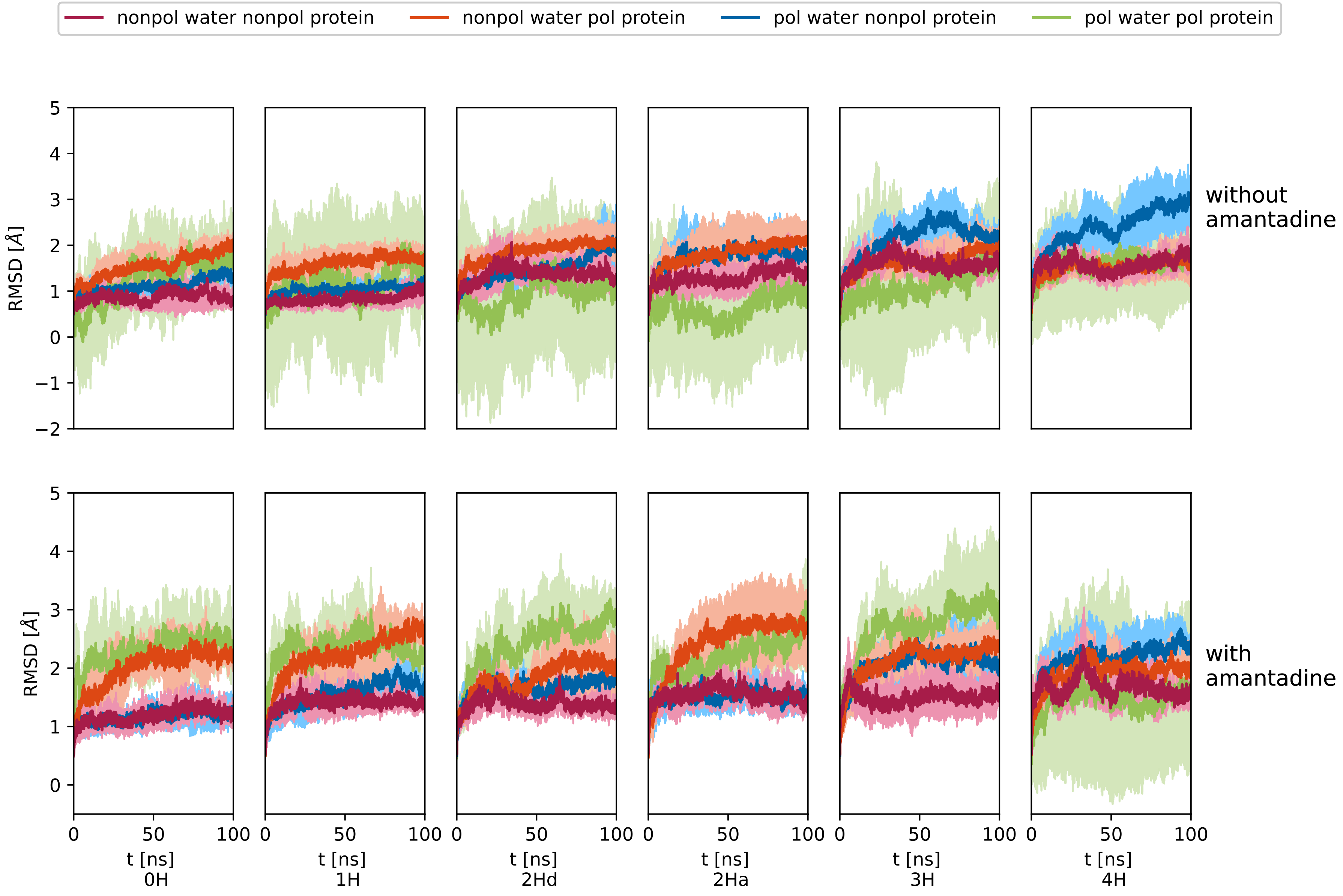


**Figure S3.** RMSD of the whole channel calculated only on the backbone. The colors indicate the polarizable treatment. Averages over all three replicas (darker lines) and standard deviation (lighter areas).

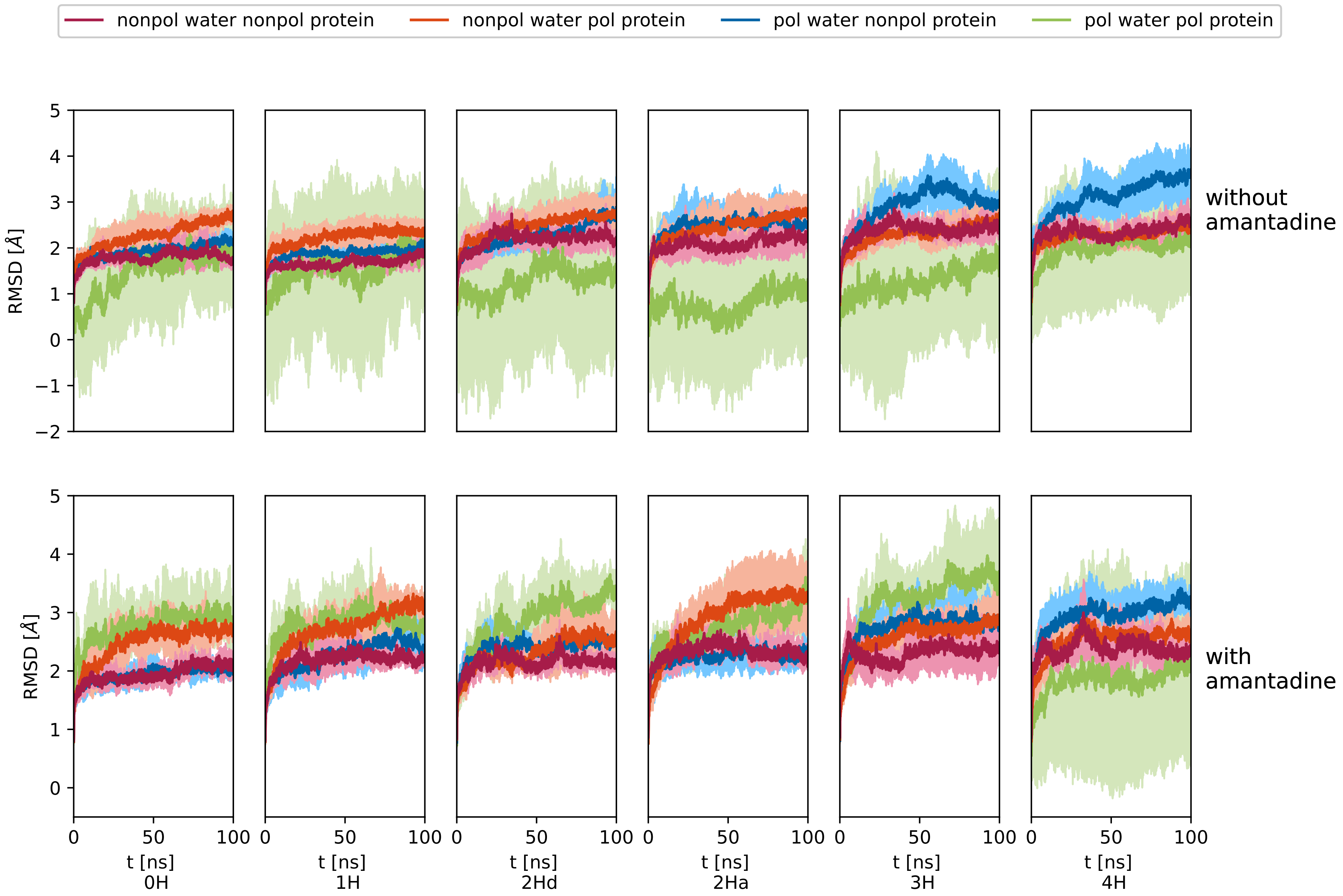


**Figure S4.** RMSD of the whole channel calculated for all atoms. The colors indicate the polarizable treatment. Averages over all three replicas (darker lines) and standard deviation (lighter areas).

## 2.2 Channel diameter

To evaluate the impact of both protonation state and force field combination, we employed heat maps based on C-C or N-N distances to facilitate direct comparisons. While a more detailed characterization of the channel structure can be obtained via residue-resolved channel diameter analysis, this approach requires computationally intensive calculations. The HOLE program (Smart et al., 1993, 1996), designed for analyzing ion channel pore dimensions, was employed for this purpose. The program utilizes a Monte Carlo simulated annealing algorithm to identify the optimal path for a variable-radius sphere to traverse the channel, enabling graphical visualization of pore dimensions.

However, full trajectory analysis was computationally prohibitive, necessitating a restriction to 100 representative snapshots. Additionally, the Drude particle-based polarizable force fields posed compatibility issues with the HOLE software. Given that graphical visualization is already implemented within the program, juxtaposing the three independent replicas proved cumbersome. Consequently, the results are presented separately in Figures S5-S7 (without amantadine) and Figures S18-S20 (with amantadine).

HOLE struggles to handle cases where the channel splits into multiple images due to periodic boundary conditions, resulting in irregular outputs, particularly in our fully polarizable systems. Additionally, the method of expanding test spheres to determine the pore radius encounters issues near the entrance and exit of the channel, yielding unphysically large radii exceeding $10\,\text{Å}$.

The results in Figures S5-S7 and Figures S18-S20 are plotted as a function of the pore coordinate as defined by HOLE. Based on measurements from the PDB structure of 3LBW, the channel has an approximate length of $30\,\text{Å}$. In Figures S5-S7 and Figures S18-S20, the center of the channel is expected to be located around $\zeta = 40\,\text{Å}$ and should extend for $15\,\text{Å}$ in both directions according to the channel length. Consequently, the computed maximum channel radius is approximately $5\,\text{Å}$, while the minimum radius is around $3\,\text{Å}$. The latter should correspond to half of our N-N distance at the tetrad, which is the case.

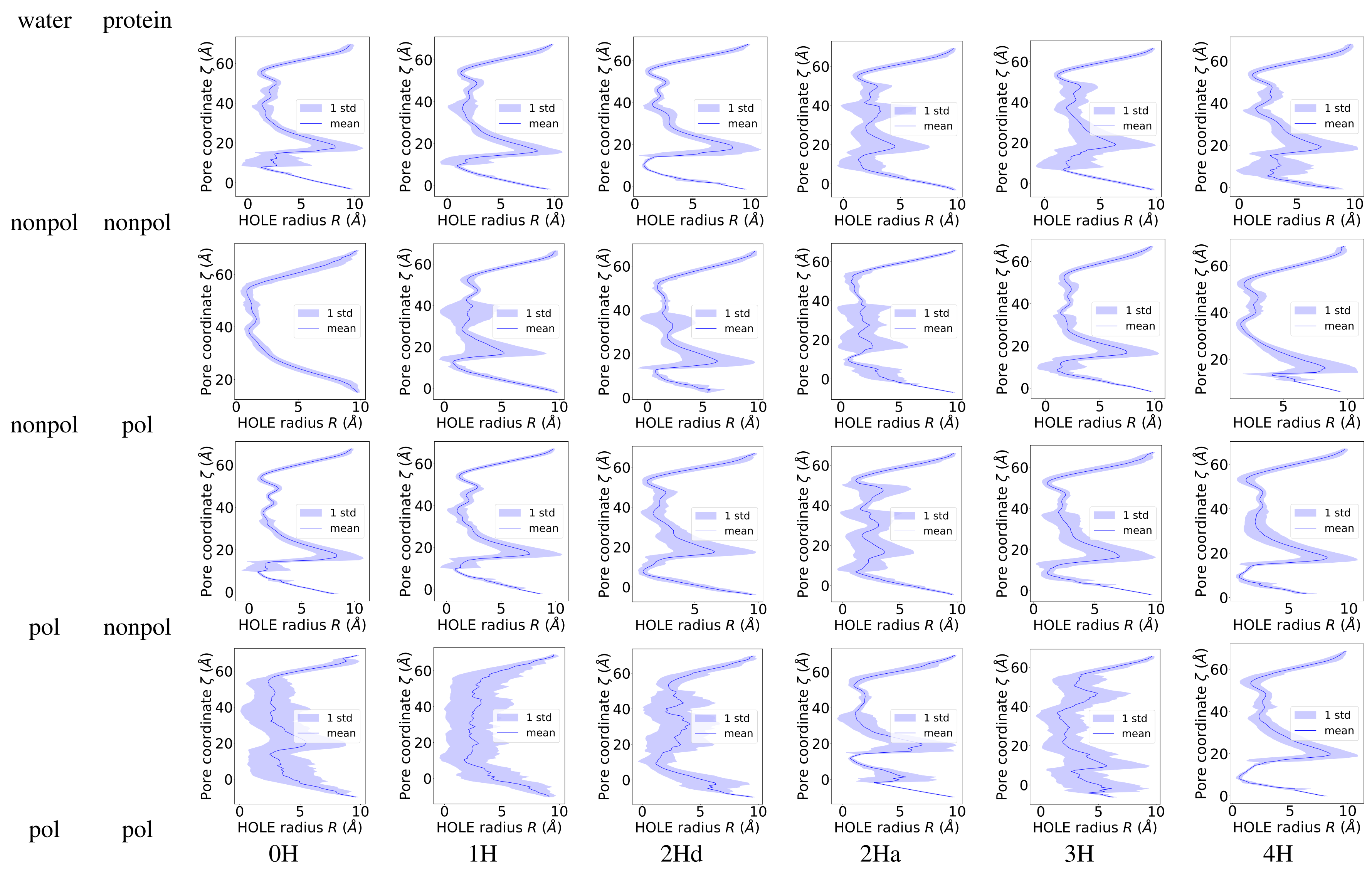


**Figure S5.** Average pore radius (dark blue) with standard deviation (light blue) in replica 1 of the simulations without amantadine.

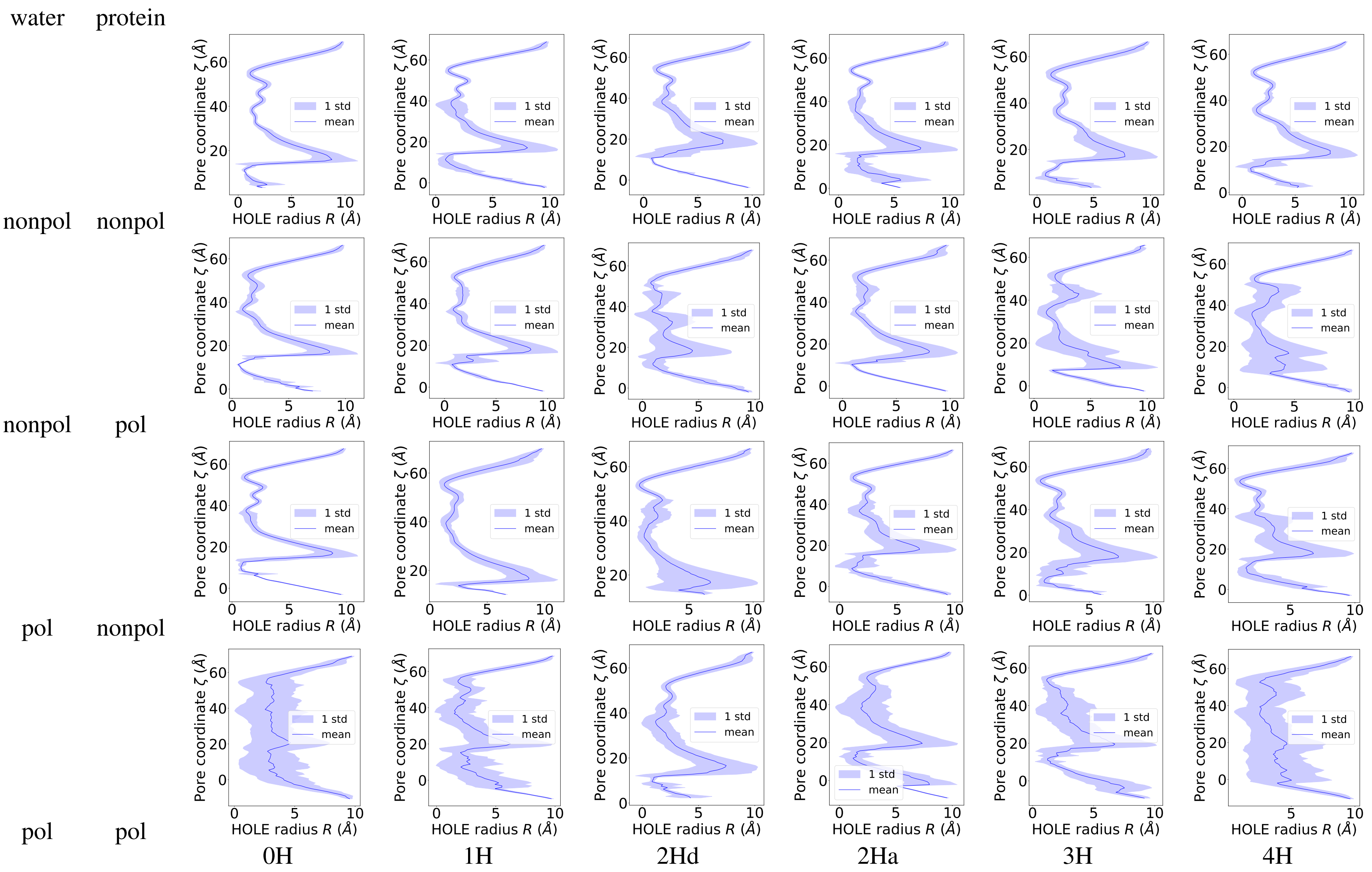


**Figure S6.** Average pore radius (dark blue) with standard deviation (light blue) in replica 2 of the simulations without amantadine.

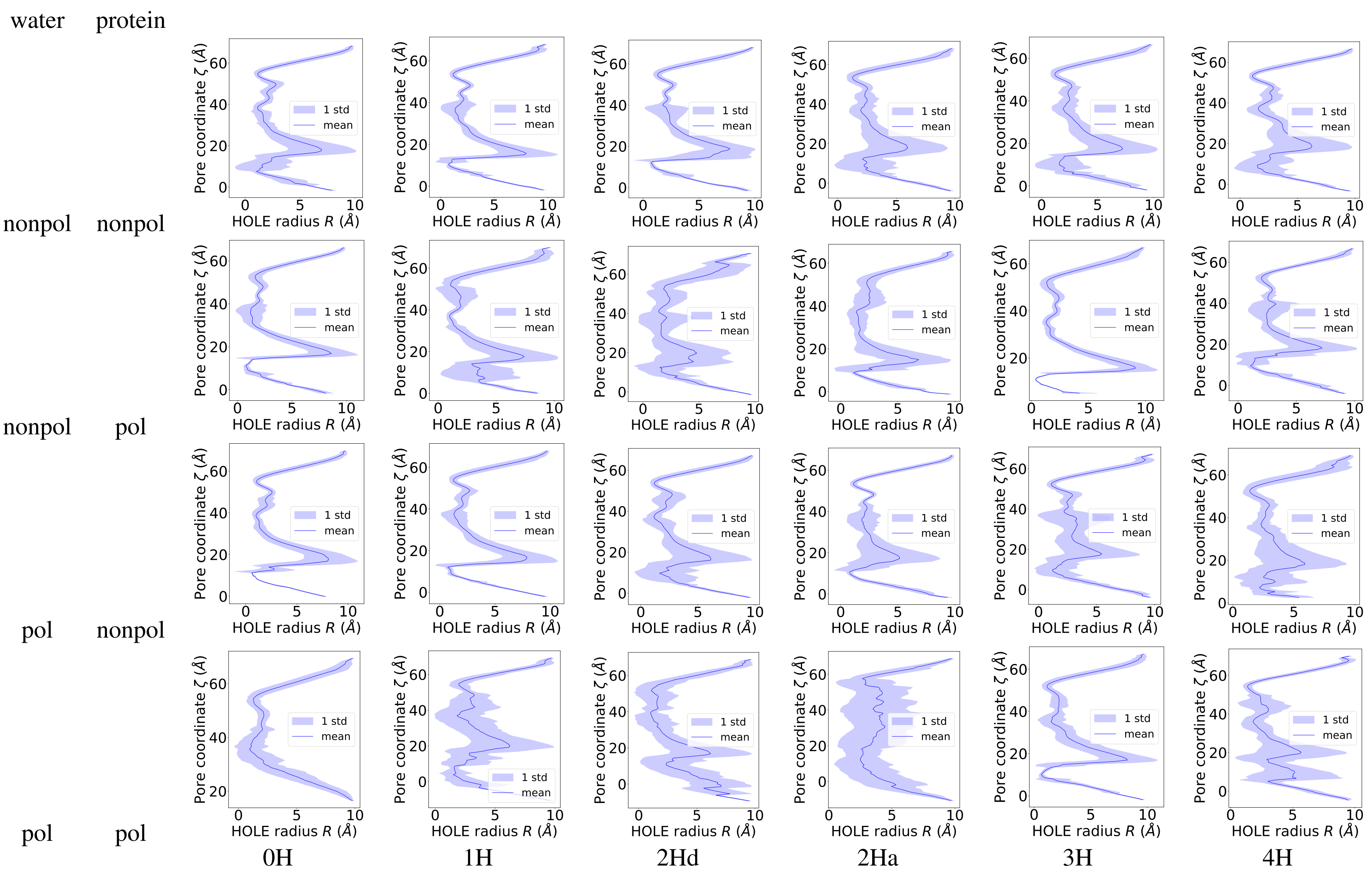


**Figure S7.** Average pore radius (dark blue) with standard deviation (light blue) in replica 3 of the simulations without amantadine.

## 2.3 Preliminary results on water diffusion

Fig. S8 presents the diffusion coefficient of water both inside and outside the ion channel. It is well-established that the self-diffusion coefficient of bulk TIP3P water is approximately twice as high as the experimentally measured value (Kumar et al., 2015), whereas SWM4 water provides a significantly more accurate representation of experimental diffusion rates (Lamoureux et al., 2006). As expected, our results confirm that the diffusion coefficient of TIP3P water is substantially higher than that of SWM4 water, but this discrepancy is most pronounced in simulations where the membrane and protein are non-polarizable. Interestingly, when a polarizable membrane and protein are included, TIP3P water exhibits reduced diffusion, bringing it closer to the diffusion behavior of SWM4 water. This suggests that introducing polarizability in the membrane and protein may alter the local water dynamics, possibly by influencing hydrogen-bond networks or restructuring hydration layers around the protein-lipid interface.

In contrast, the diffusion coefficient of SWM4 water remains largely unaffected by whether the protein and membrane are polarizable or non-polarizable. This robustness suggests that SWM4 water, due to its intrinsic polarizability, already incorporates effects that make it less sensitive to environmental perturbations caused by protein or membrane polarization. These findings highlight the intricate interplay between water dynamics and force field parameters, emphasizing the importance of selecting appropriate water models based on the level of membrane and protein polarizability when simulating ion channels. The diffusion

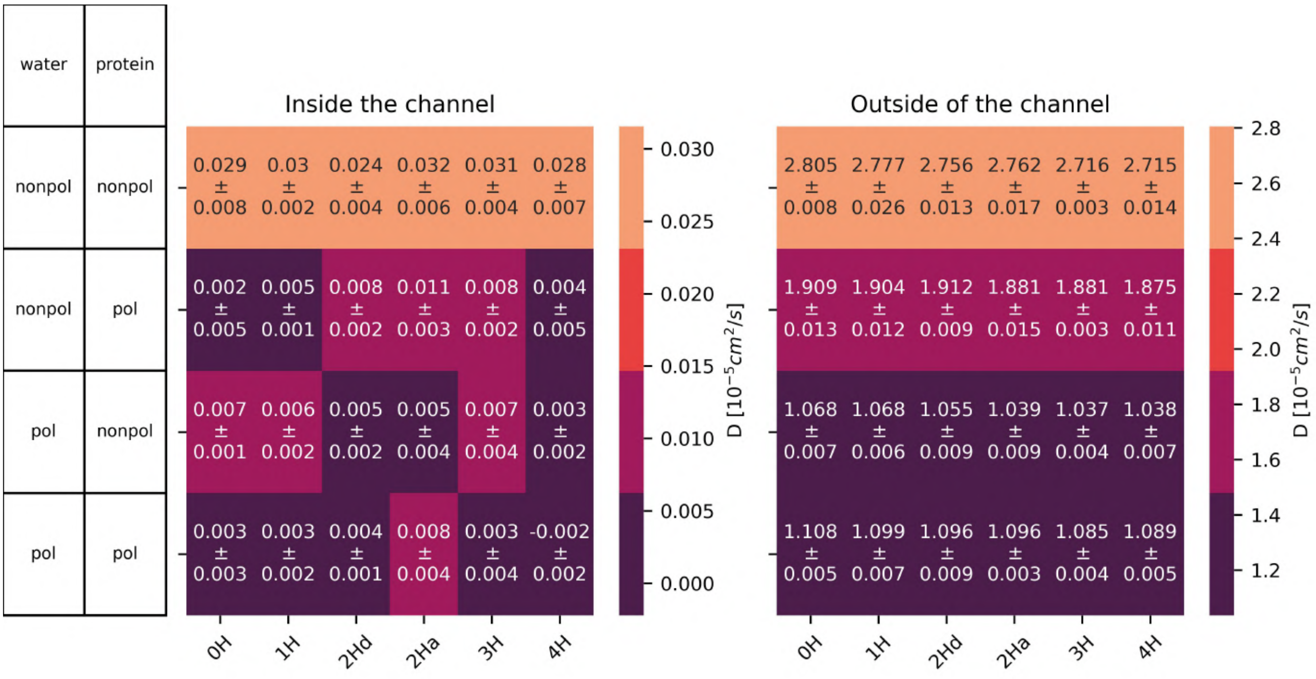


**Figure S8.** Diffusion coefficient of water inside (left) and outside (right) of the channel.

coefficients of water are expected to undergo significant changes once our proton transfer water model is fully developed. In this model, proton hopping mechanisms, which give rise to the formation of oxonium ($H_3O^+$) and hydroxide ($OH^-$) ions, will play a crucial role in influencing water dynamics. Unlike standard non-reactive water models, which treat water diffusion as a purely translational process, proton transfer introduces an additional Grotthuss mechanism, where charge transport occurs via continuous hydrogen-bond rearrangement. This effect can substantially alter the local and bulk diffusion behavior.

Given these anticipated modifications, the current diffusion coefficient results should be regarded as preliminary. The inclusion of a proton transfer model is expected to provide a more realistic representation of water behavior in confined environments such as ion channels, where proton dynamics are known to have a profound effect on hydration structure, local electric fields, and overall transport properties. Future studies incorporating this model will offer deeper insights into the complex interplay between proton transport, water mobility, and membrane/protein interactions.

## 2.4 Water penetration

Fig. S9 shows the number of water molecules in the channel. For each amino acid of the channel, the number of water molecules at the same z coordinate is displayed. The two terminal amino acids at both ends of the channel were disregarded, to focus on water that is strictly inside the channel. The trends are very similar in all cases: the number of water molecules around the bottleneck increases with increasing protonation of His37. The total number of water molecules in the channel is shown in Fig. S10. It increases steadily with the protonation state. In general, the systems with non-polarizable protein show a smaller amount of water in the channel, which could also lead to the difference in the radial distribution functions depicted in the manuscript. If there are fewer water molecules around His37, they can all coordinate via hydrogen bonds, and the second shell could get depleted.

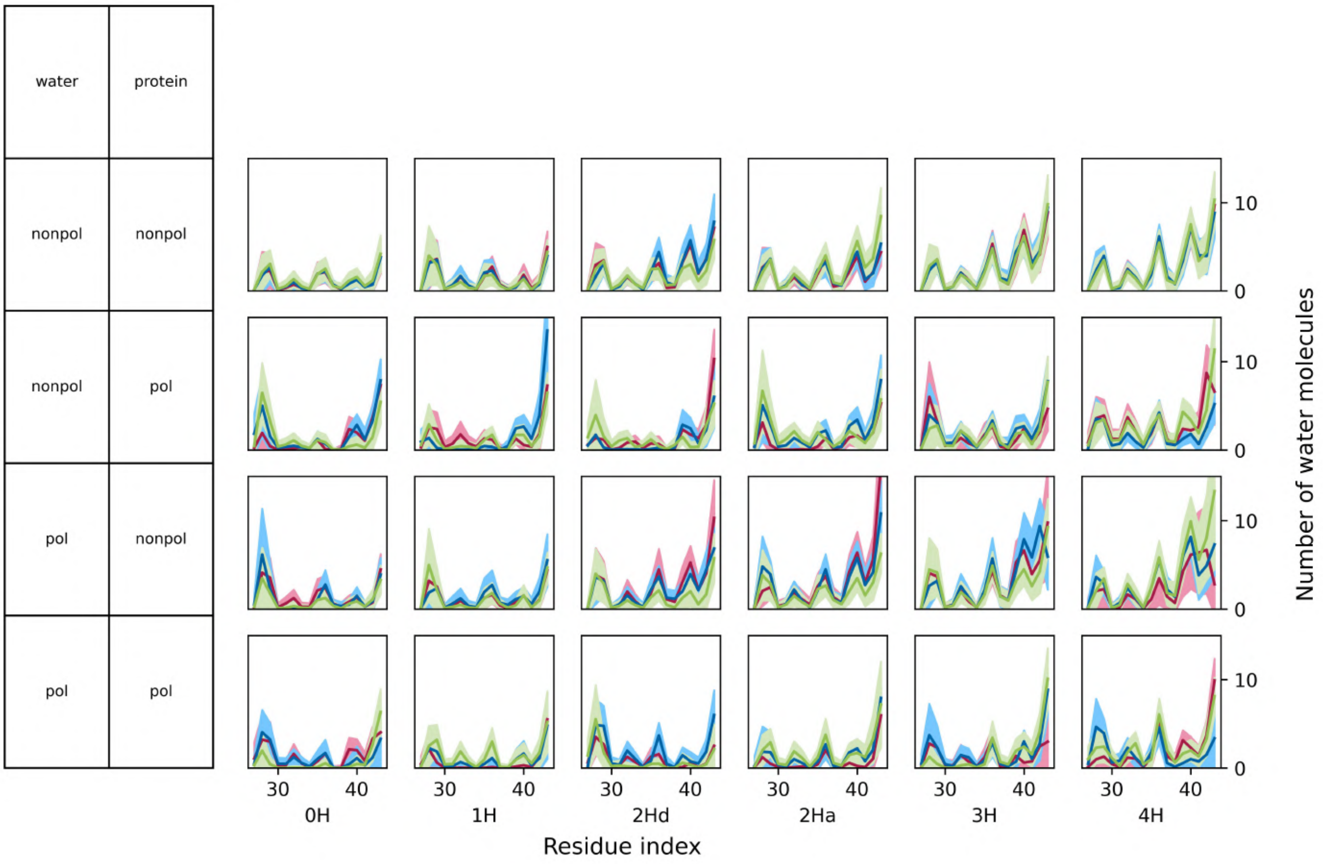


**Figure S9.** Number of water molecules in the channel in the simulations without amantadine as a function of the residue index of the amino acids. The three replicas are distinguished by the colors (red, green, and blue). The solid lines show the average, and the lightly colored areas show one standard deviation for the replica.

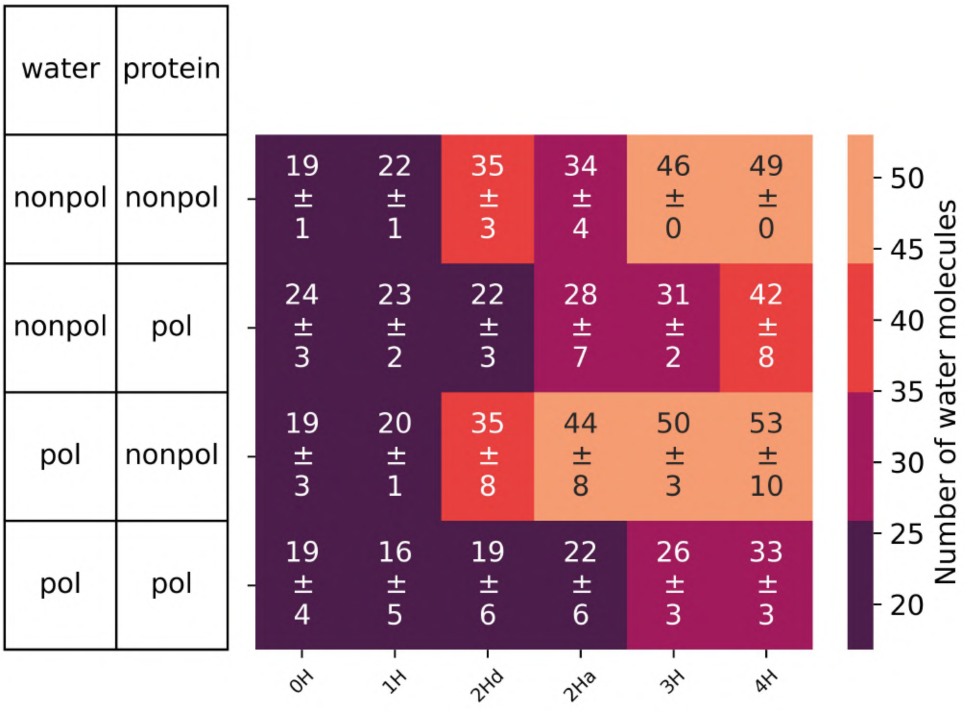


**Figure S10.** Total number of water molecules in the channel in the simulations without amantadine (average over the three replicas and standard deviation).

## 2.5 Effect of amantadine

Fig. S11 presents the diffusion coefficient of amantadine using the various force field combinations and the various protonation states of the histidine tetrad. Similarly to the trends observed for water diffusion, the highest diffusion rates occur when both the protein and water are modeled as non-polarizable. However, even in this scenario, the diffusion coefficient remains significantly low across all cases, suggesting that amantadine is relatively immobilized within the channel environment. This observation reinforces the idea that amantadine maintains a stable binding position, likely due to its interactions with the surrounding protein and membrane environment.

The reduced mobility of amantadine may be attributed to a combination of hydrophobic interactions, electrostatic stabilization, and steric hindrance within the channel. A more detailed analysis of the contacts between amantadine and M2, determined with LigPlot+ 2.2 (Wallace et al., 1995; Laskowski and Swindells, 2011) using 100 snapshots of the trajectory, is shown in Figures S12-S15. Besides the hydrogen bonds shown in the manuscript (mainly to Ala30, Ser31 and Val27), several hydrophobic contacts have been identified, mainly to Ala30, Ser31, Val27, and Gly34. Strong binding interactions between amantadine and the protein may lead to extended residence times, while steric constraints within the pore could further restrict significant translational movement. Additionally, the introduction of polarizable force fields appears to enhance local electrostatic interactions, further stabilizing the molecule in its preferred position. This result is consistent with previous studies suggesting that amantadine acts as a channel blocker, preventing ion permeation by occupying a well-defined binding site.

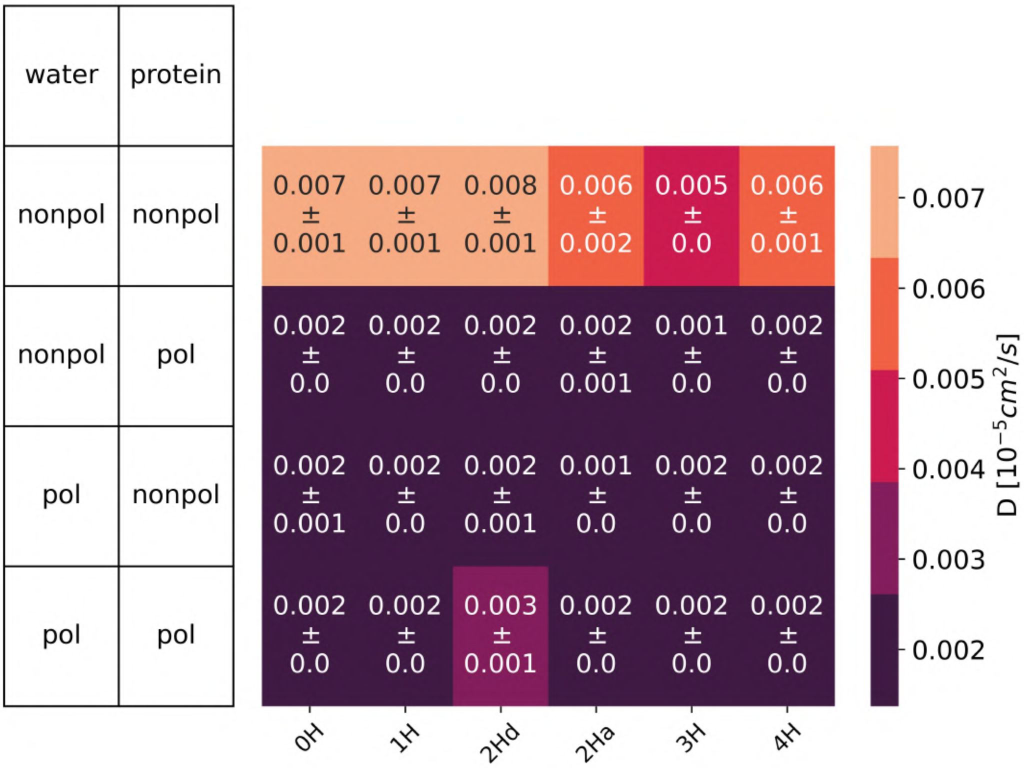


**Figure S11.** Diffusion coefficient of amantadine.

Given the consistently low diffusion coefficients observed in all cases, it is likely that amantadine remains structurally confined and does not undergo rapid lateral diffusion within the channel. Future investigations involving free energy calculations and longer simulation timescales will be necessary to gain a deeper understanding of the binding kinetics and residence time of amantadine, particularly in the presence of different protonation states and membrane environments.

The number of water molecules in the channel with amantadine is shown in Figures S16 and S17. The trends are very similar to the simulations without amantadine: the amount of water increases with an increasing protonation state. There is altogether more water in the channel with amantadine, which might be explained by the slightly larger interhelix distances. However, it must be noted that this increased amount of water by no means corresponds to an increased transport through the channel. Without a concentration gradient or other external driving force, the channel can fill up with water from both ends in the simulation by simple diffusion, which is not what happens in a complete virus.

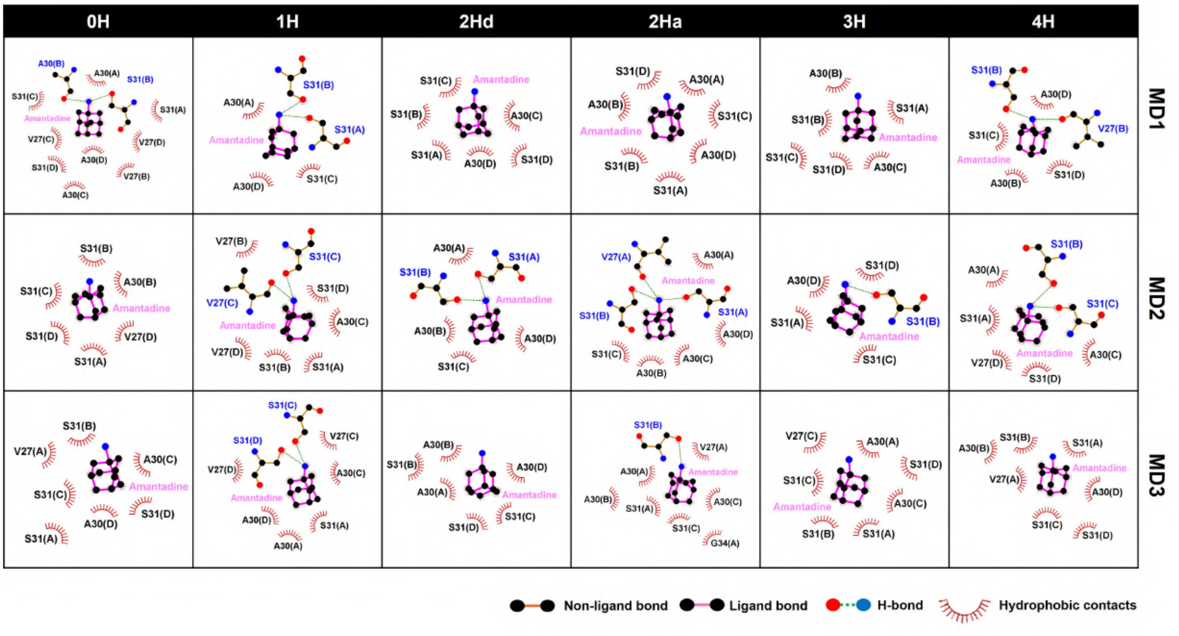


**Figure S12.** Contacts between amantadine and the nonpolarizable protein for all protonation states with the TIP3P water model.

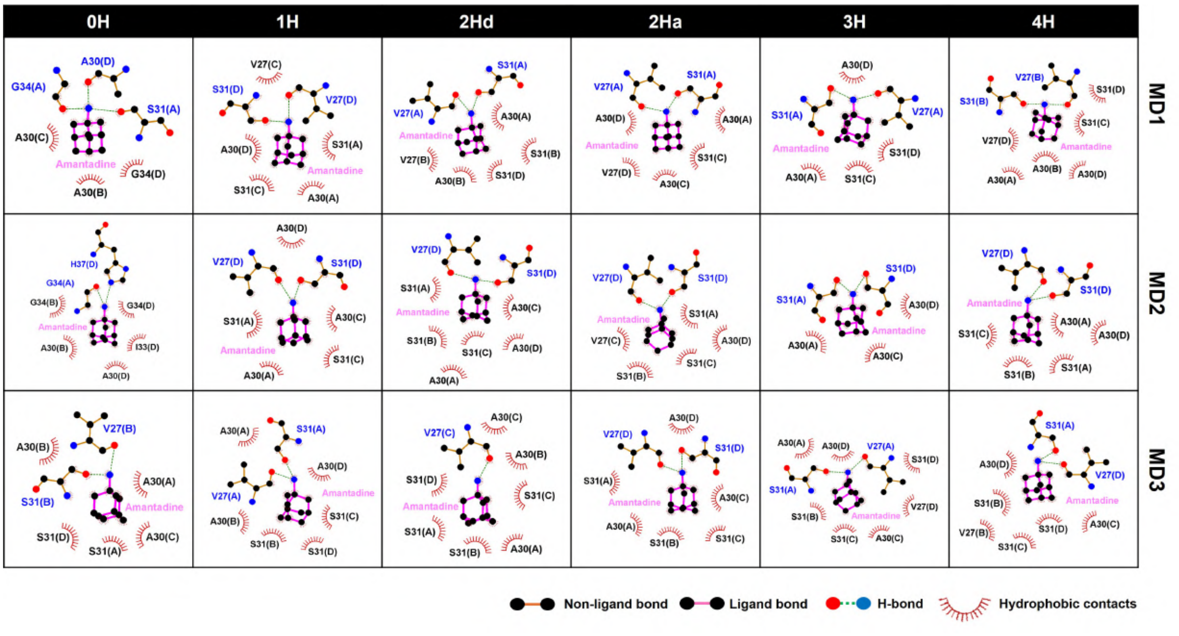


**Figure S13.** Contacts between amantadine and the polarizable protein for all protonation states with the TIP3P water model.

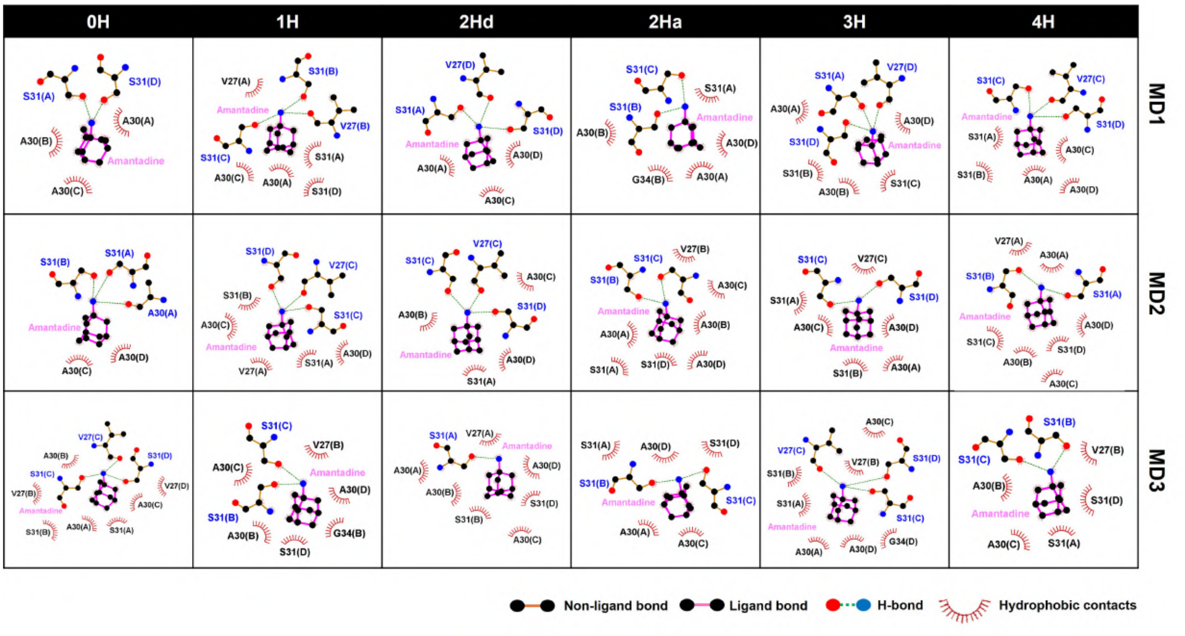


**Figure S14.** Contacts between amantadine and the nonpolarizable protein for all protonation states with the SWM4 water model.

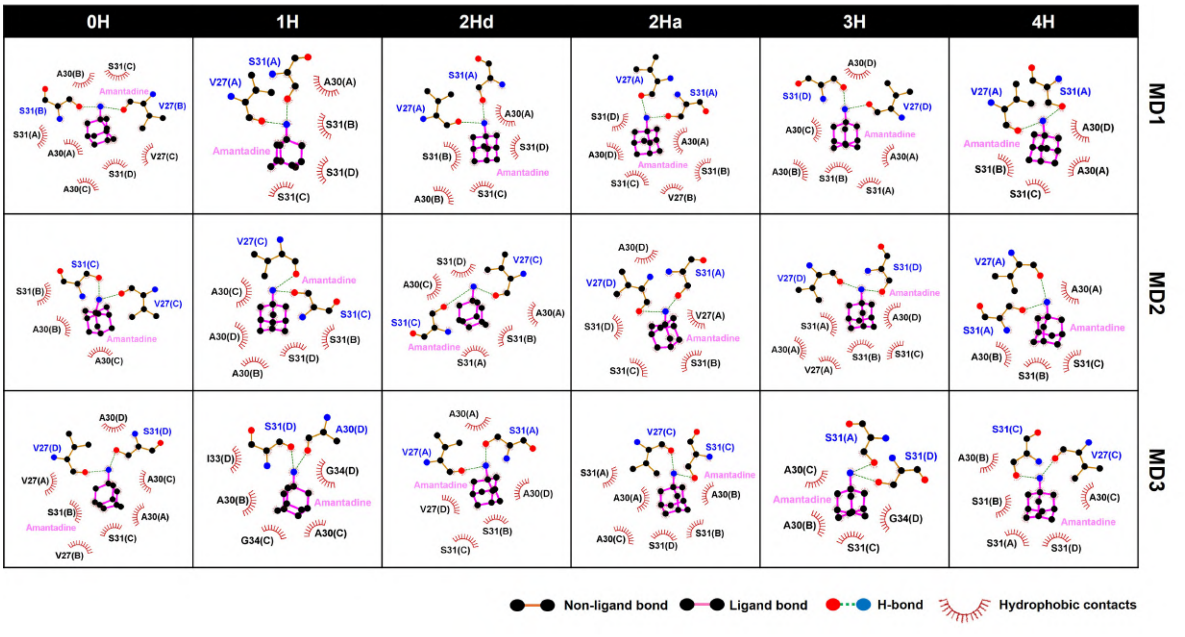


**Figure S15.** Contacts between amantadine and the polarizable protein for all protonation states with the SWM4 water model.

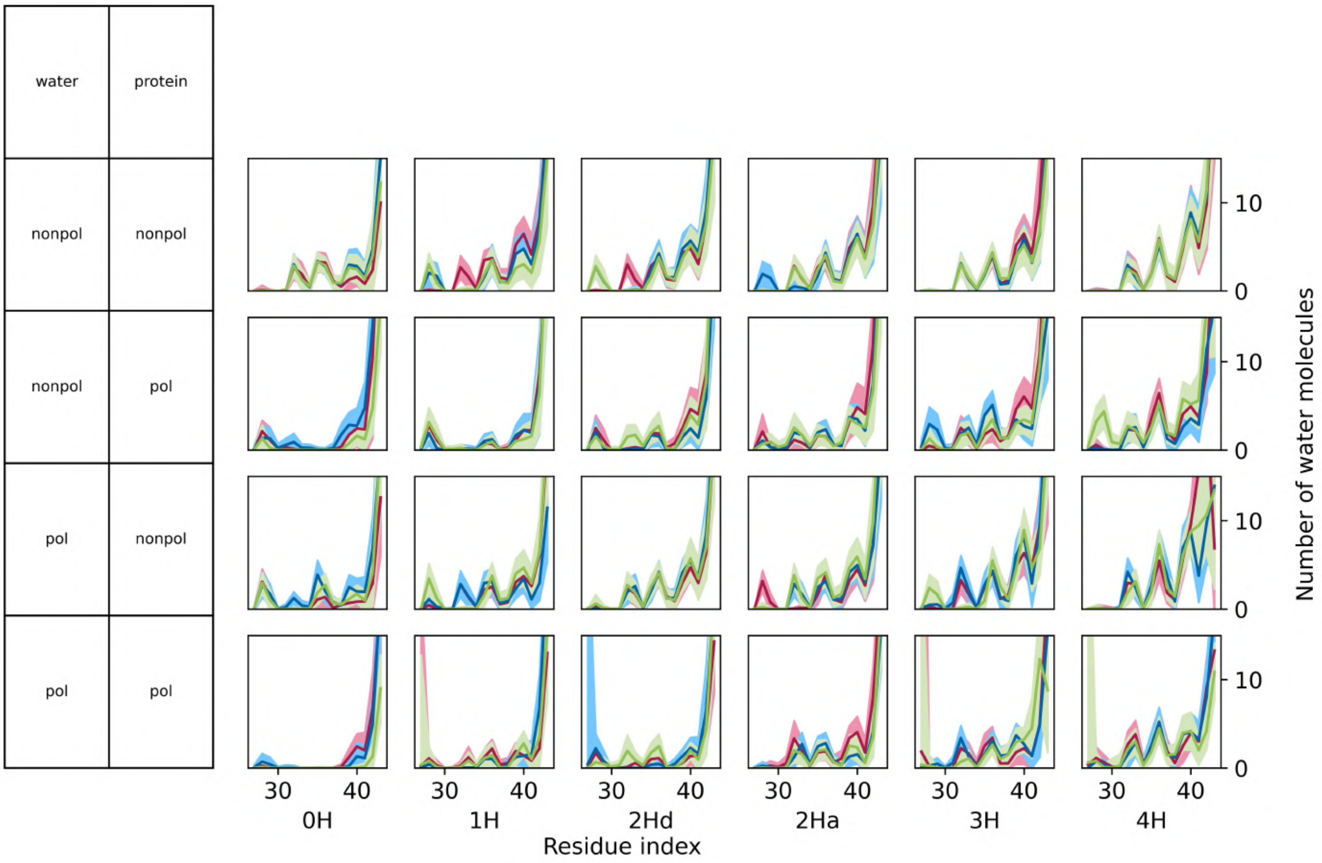


**Figure S16.** Number of water molecules in the channel in the simulations with amantadine as a function of the residue index of the amino acids. The three replicas are distinguished by the colors (red, green, and blue). The solid lines show the average, and the lightly colored areas show one standard deviation for the replica.

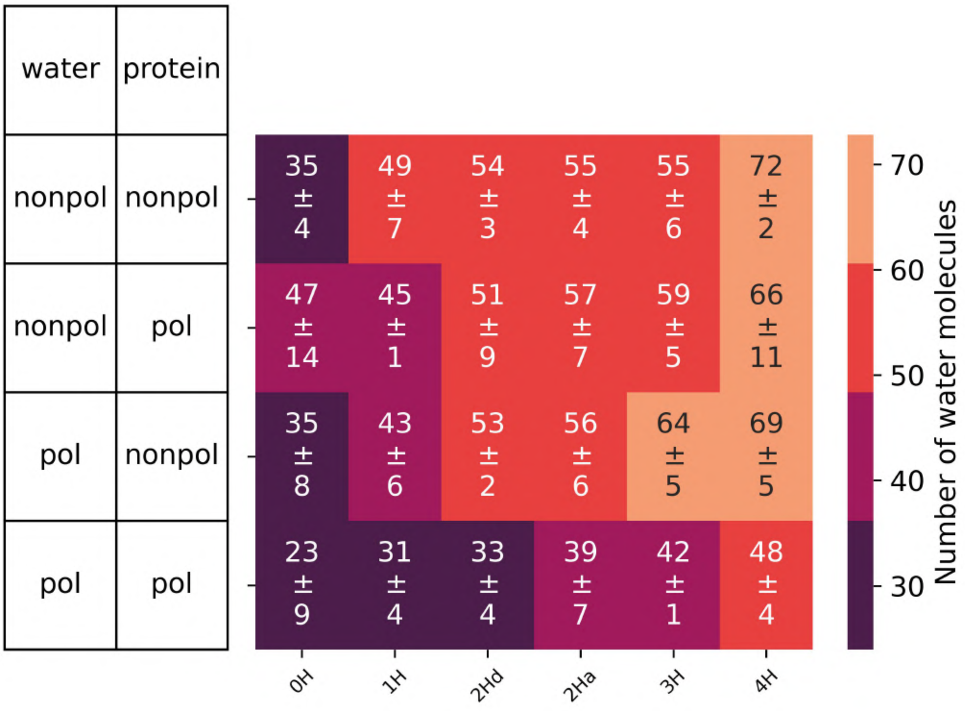


**Figure S17.** Total number of water molecules in the channel in the simulations with amantadine (average over the three replicas and standard deviation).

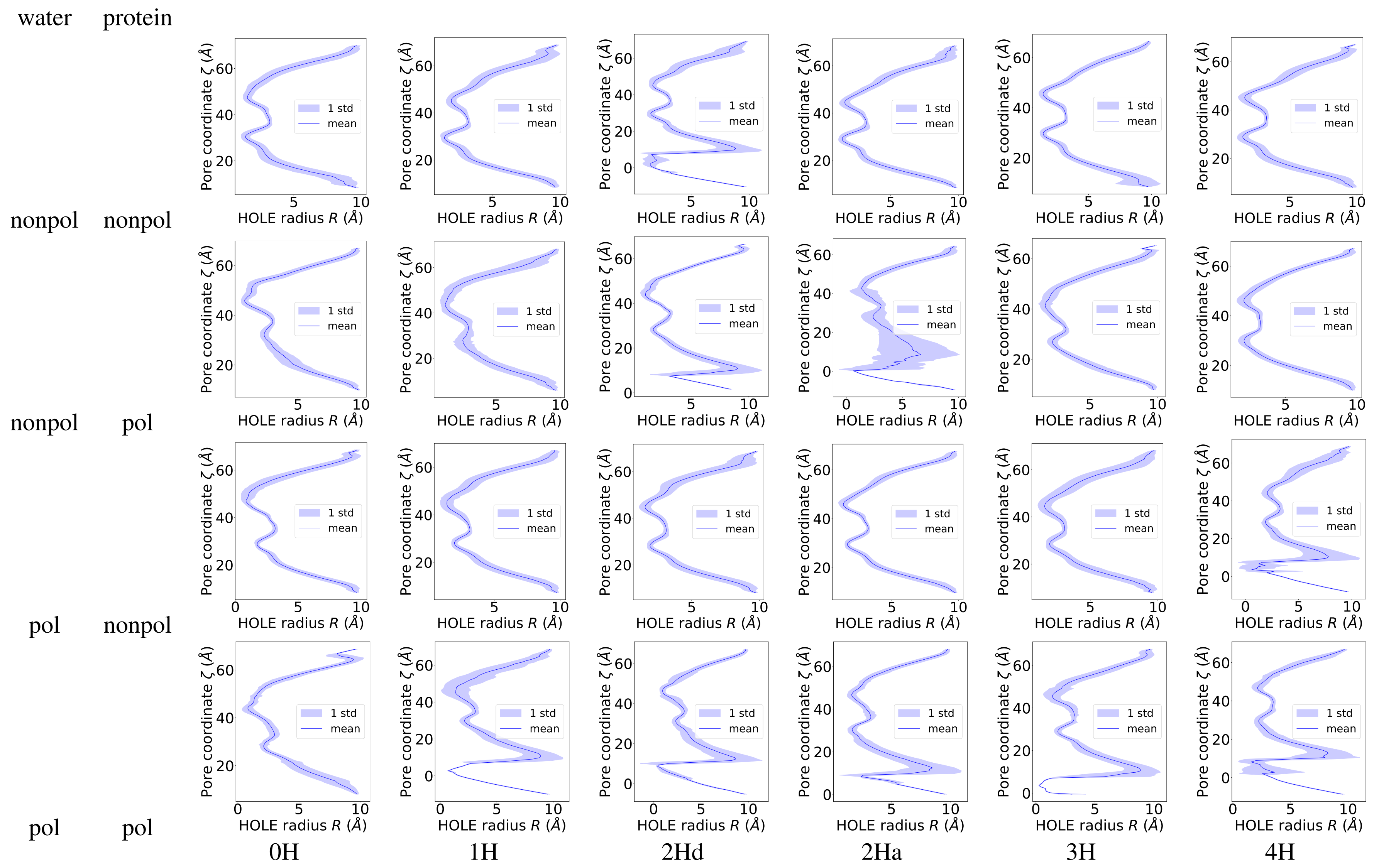


**Figure S18.** Average pore radius (dark blue) with standard deviation (light blue) in replica 1 of the simulations with amantadine.

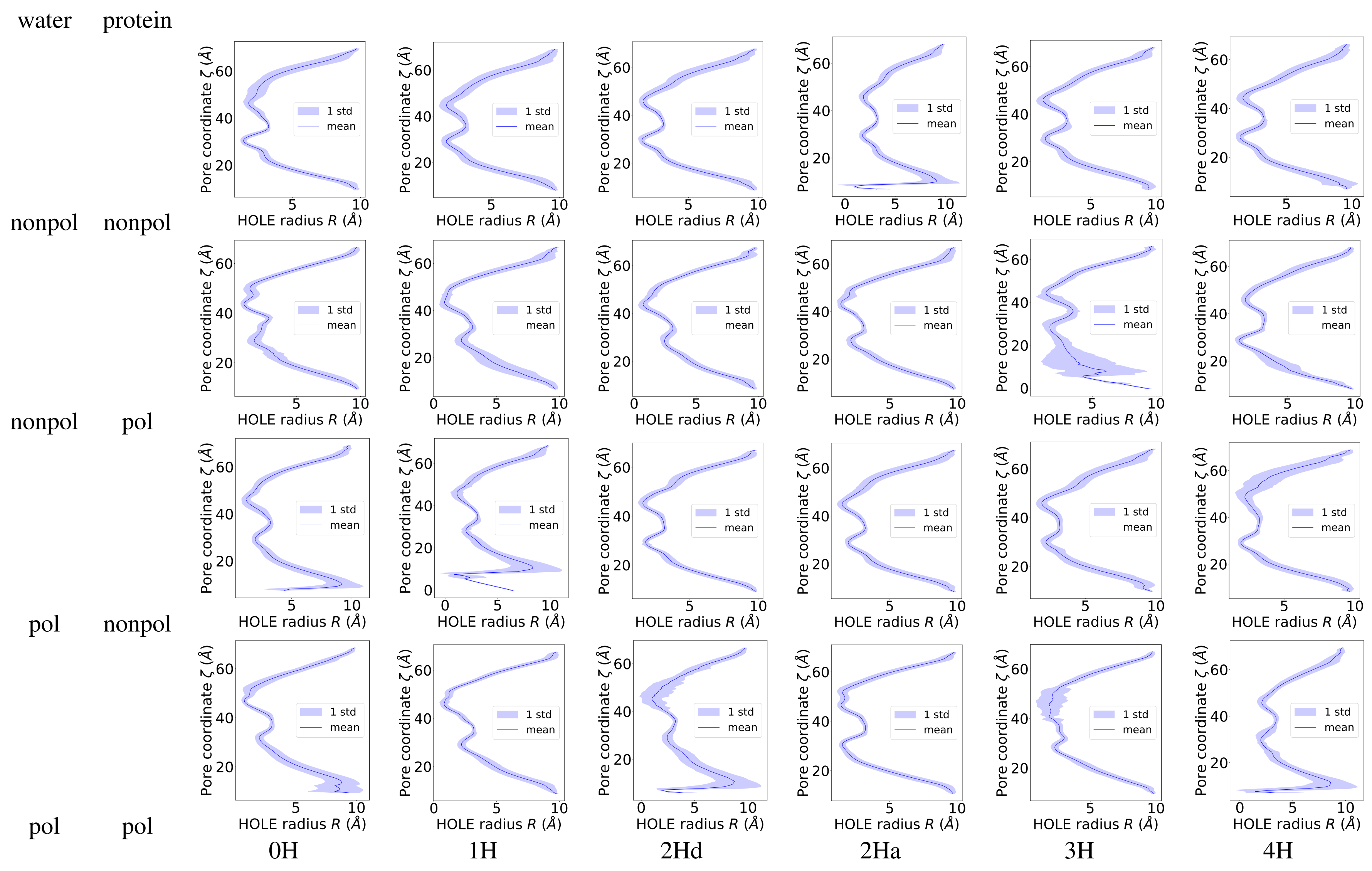


**Figure S19.** Average pore radius (dark blue) with standard deviation (light blue) in replica 2 of the simulations with amantadine.

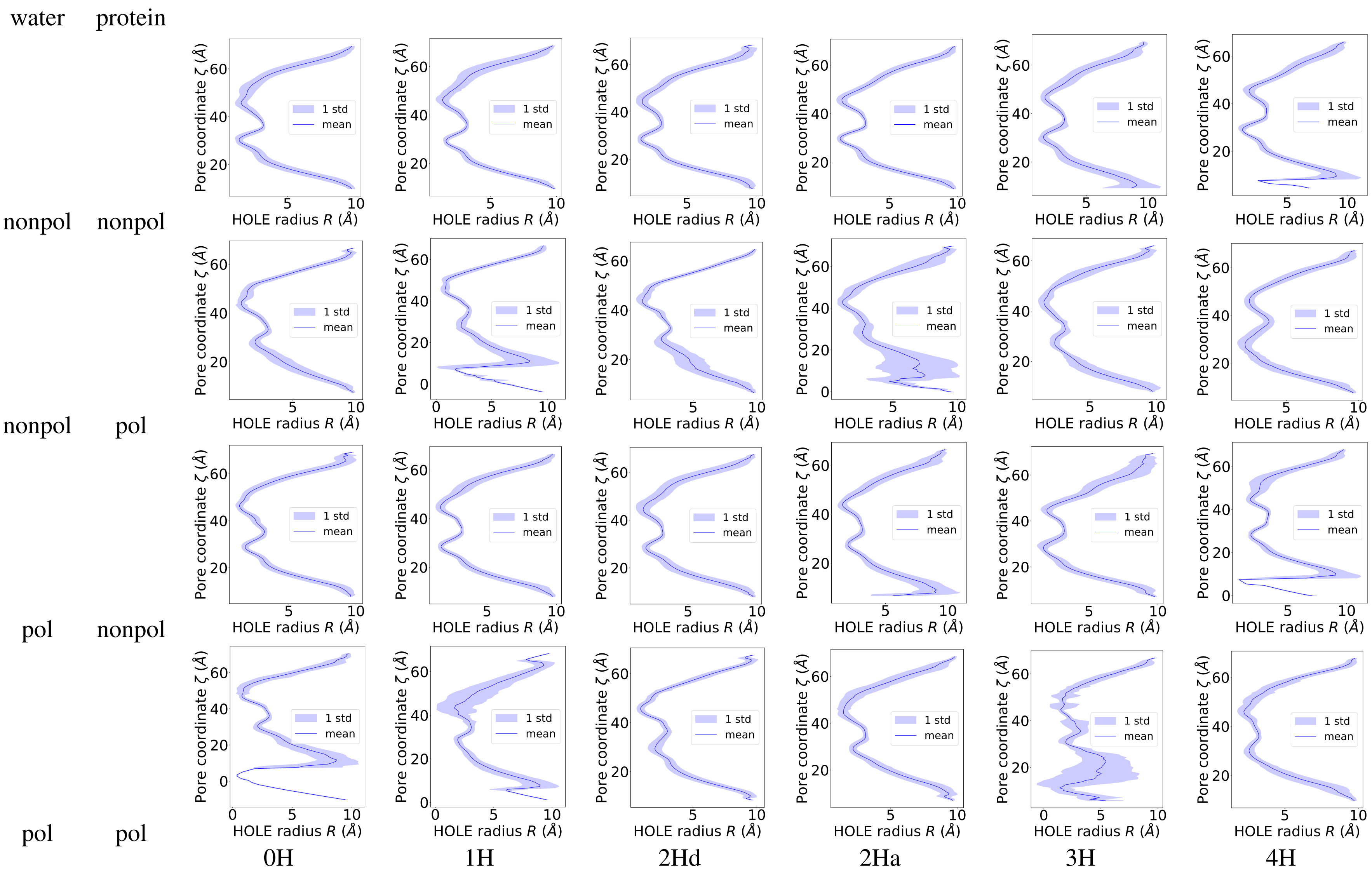


**Figure S20.** Average pore radius (dark blue) with standard deviation (light blue) in replica 3 of the simulations with amantadine.

## REFERENCES


[Dataset] Acharya, R., Polishchuk, A., and DeGrado, W. (2010). High Resolution Crystal Structure of Transmembrane Domain of M2. doi:10.2210/pdb3lbw/pdb

Kumar, H., Dasgupta, C., and Maiti, P. K. (2015). Structure, dynamics and thermodynamics of single-file water under confinement: effects of polarizability of water molecules. *RSC Adv.* 5, 1893–1901

Kuzmanic, A. and Zagrovic, B. (2010). Determination of ensemble-average pairwise root mean-square deviation from experimental b-factors. *Biophys. J.* 98, 861–871

Lamoureux, G., Harder, E., Vorobyov, I. V., Roux, B., and MacKerell, A. D. (2006). A Polarizable Model of Water for Molecular Dynamics Simulations of Biomolecules. *Chem. Phys. Lett.* 418, 245–249. doi:10.1016/j.cplett.2005.10.135

Laskowski, R. A. and Swindells, M. B. (2011). LigPlot+: multiple ligand-protein interaction diagrams for drug discovery. *J. Chem. Inf. Model.* 51, 2778–2786

[Dataset] Nishimura, K., Kim, S., Zhang, L., and Cross, T. (2003). The Closed State Structure of M2 Protein $H^+$ Channel by Solid State NMR Spectroscopy. doi:10.2210/pdb1nyj/pdb

[Dataset] Sharma, M., Yi, M., Dong, H., Qin, H., Peterson, E., Busath, D., et al. (2010). Solid State NMR Structure of the M2 Proton Channel from Influenza A Virus in Hydrated Lipid Bilayer. doi:10.2210/pdb2l0j/pdb

Smart, O. S., Goodfellow, J. M., and Wallace, B. A. (1993). The pore dimensions of gramicidin a. *Biophys. J.* 65, 2455–2460

Smart, O. S., Neduvelil, J. G., Wang, X., Wallace, B. A., and Sansom, M. S. (1996). HOLE: a program for the analysis of the pore dimensions of ion channel structural models. *J. Mol. Graph.* 14, 354–60, 376

Wallace, A. C., Laskowski, R. A., and Thornton, J. M. (1995). LIGPLOT: a program to generate schematic diagrams of protein-ligand interactions. *Protein Eng. Des. Sel.* 8, 127–134
